# Dynamic Copper(I) and Silver(I) Complexes of Tri‐*tert*‐butyl‐cyclotriphosphane

**DOI:** 10.1002/chem.202500746

**Published:** 2025-04-17

**Authors:** Toni Grell, Peter Wonneberger, Divine Mbom Yufanyi, Peter Lönnecke, Evamarie Hey‐Hawkins

**Affiliations:** ^1^ Dipartimento di Chimica, Università degli Studi di Milano, Via Camillo Golgi 19 Milan Italy; ^2^ Faculty of Chemistry and Mineralogy, Leipzig University, Johannisallee 29 Leipzig Germany; ^3^ Department of Fundamental Science, Higher Technical Teacher Training College Bambili The University of Bamenda P.O. Box 39 Bambili Bamenda Cameroon; ^4^ Present Address: Faculty of Chemistry and Mineralogy Institute of Bioanalytical Chemistry Leipzig University Deutscher Platz 5 Leipzig Germany; ^5^ Faculty of Chemistry and Chemical Engineering, Department of Chemistry Babes‐Bolyai University 1, Kogalniceanu str. Cluj‐Napoca Romania

**Keywords:** copper, dynamic complexes, equilibria, oligophosphanes, silver

## Abstract

The reaction of *cyclo*‐(P_3_ *t*Bu_3_) with Cu^I^ and Ag^I^ salts led to the di‐ and tetranuclear complexes [Cu_*n*_(μ‐Br)_*n*_{μ‐*cyclo*‐(P_3_ *t*Bu_3_)‐κ*P* ^1^,κ*P* ^2^}_2_] with *n* = 2 (**1**) and *n* = 4 (**2**) and [Ag*_n_* (OTf)*_n_* (CH_3_CN)_2_ *_n_*_−2_{μ‐*cyclo*‐(P_3_ *t*Bu_3_)‐κ*P* ^1^,κ*P* ^2^}_2_] with *n* = 2 (**4**) and *n* = 4 (**5**). Complexes **1**, **2**, **4** and **5** were isolated as crystalline powders or single crystals and characterized by X‐ray diffraction. In solution, these complexes exhibit a dynamic equilibrium which involves continuous dissociation and reforming of phosphorus−metal bonds leading to isomeric intermediates. This behavior was demonstrated by low‐temperature ^31^P{^1^H} NMR spectroscopy and the conclusions were corroborated by DFT calculations.

## Graphical abstract

The reaction of *cyclo*‐(P_3_ *t*Bu_3_) with Cu^I^ and Ag^I^ salts led to the di‐ and tetranuclear complexes [Cu*_n_* (μ‐Br)*_n_* {μ‐*cyclo*‐(P_3_ *t*Bu_3_)‐κ*P* ^1^,κ*P* ^2^}_2_] and [Ag*_n_* (OTf)*_n_* (CH_3_CN)_2_ *_n_*_−2_{μ‐*cyclo*‐(P_3_ *t*Bu_3_)‐κ*P* ^1^,κ*P* ^2^}_2_] (*n* = 2, 4). In solution, these complexes exhibit a dynamic equilibrium involving continuous dissociation and reforming of phosphorus‐metal bonds. This behavior was demonstrated by low‐temperature ^31^P{^1^H} NMR spectroscopy and corroborated by DFT calculations.

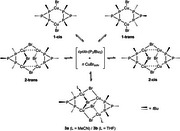

## 1 Introduction

Phosphorus‐rich compounds represent a class of substances with a long tradition.^[^ ^1^, ^2^ ^]^ While having been for most of the time an academic curiosity, in recent years these compounds have gained significance as they play an increasingly important role in the generation of phosphorus‐rich metal phosphides MP*_x_* (*x* > 1),^[^ ^3^ ^]^ the activation of white phosphorus and its direct conversion to phosphanes^[^ ^4^ ^]^ as well as catalysis of industrially relevant processes.^[^ ^5^ ^]^

The coordination chemistry of phosphorus‐rich ligands is particularly intriguing, as they possess many donor atoms. While many complexes with anionic phosphorus‐rich ligands have been published,^[^ ^6^, ^7^, ^8^ ^]^ fewer examples of coordination compounds with neutral mono‐ and polycyclic oligophosphanes are known,^[^ ^9^, ^10^, ^11^, ^12^ ^]^ but show a fascinating diversity as well. A peculiar feature of these complexes is dynamic behavior in solution. The gold(I) complexes of hexa‐*tert*‐butyl‐octaphosphane, [Au*_n_* Cl*_n_* ({*cyclo*‐(P_4_ *t*Bu_3_)}_2_)] (*n* = 1–3) for example show two types of an intramolecular rearrangement process where the {AuCl} unit migrates between phosphorus donor atoms.^[^ ^13^ ^]^ Another example are the gold(I) complexes of one of the simplest *cyclo*‐phosphanes, *cyclo*‐(P_4_ *t*Bu_4_), where {AuCl} units are constantly exchanged between *cyclo*‐tetraphosphane molecules.^[^ ^14^ ^]^

**Scheme 1:**
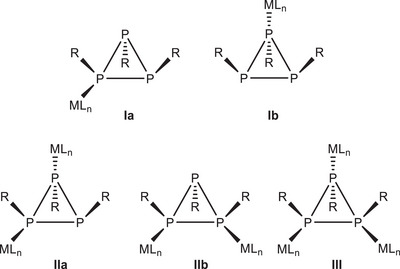
Possible bonding modes of *cyclo*‐(P_3_R_3_) in metal complexes.

A number of metal complexes which contain monocyclic triphosphanes *cyclo*‐(P_3_R_3_) are known.^[^ ^15^ ^]^ Theoretically, five different bonding modes can be expected, depending on the number of coordinated metal complex fragments, namely two types of mono‐ (κ*P* ^1^) and bis‐monodentate (κ*P* ^1^,κ*P* ^2^) bonding modes, due to the different orientation of the three R groups (*cis* or *trans*), and one tris‐monodentate (κ*P* ^1^,κ*P* ^2^,κ*P* ^3^) bonding mode (Scheme 1). The molecular structures of several transition metal carbonyl complexes with monodentate *cyclo*‐(P_3_R_3_) ligands, for example, [M(CO)_5_{*cyclo*‐(P_3_R_3_)‐κ*P* ^1^}] (M = Cr, W, R = *i*Pr, *t*Bu, Ph),^[^ ^17^, ^18^, ^19^, ^20^ ^]^ [Cr(CO)_5_{*cyclo*‐(P_3_R_3_)‐κ*P* ^1^}] (R = 2,4,6‐Me_3_C_6_H_2_, Mes),^[^ ^20^, ^21^ ^]^ [Cr(CO)_4_{*cyclo*‐(P_3_ *i*Pr_3_)‐κ*P* ^1^}_2_],^[^ ^22^ ^]^ and [CpMn(CO)_2_{*cyclo*‐(P_3_Ph_3_)‐κ*P* ^1^}],^[^ ^19^ ^]^ the bis‐monodentate complex [{Cr(CO)_5_}_2_{*cyclo*‐(P_3_R_3_)‐κ*P* ^1^,κ*P* ^2^}] (R = Cy),^[^ ^23^ ^]^ and the tris‐monodentate 4‐methyl‐1,2,6‐triphosphatricyclo[2.2.1.0^2,6^]heptane (P_3_‐nortricyclane) complex [{Cr(CO)_5_}_3_{CH_3_C(CH_2_P)_3_‐κ*P* ^1^,κ*P* ^2^,κ*P* ^3^}],^[^ ^24^ ^]^ have been reported. Compounds, in which the three‐membered ring has been cleaved, are also known.^[^ ^25^ ^]^ Remarkably, in all known bis‐monodentate complexes only bonding mode **IIa** has been realized with the organic moieties at coordinating P atoms in *trans* arrangement. We here report reactions between *cyclo*‐triphosphane *cyclo*‐(P_3_ *t*Bu_3_) and copper(I) and silver(I) salts which resulted in dynamic multimetallic complexes.

## 2 Results and Discussion

### 2.1 Dinuclear Copper Complex

**Scheme 2:**
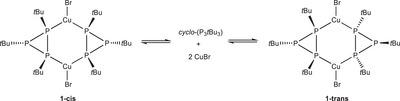
Formation of the two isomers of complex **1** in solution.

**Figure 1:**
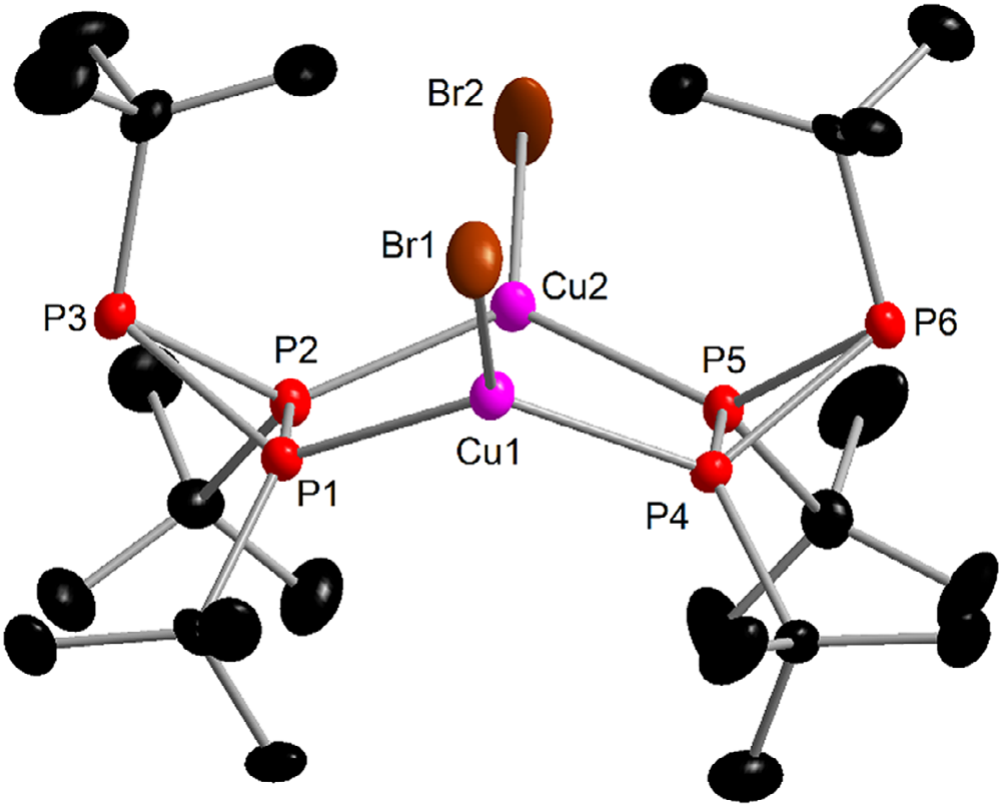
Molecular structure of complex **1**. Only one of three independent molecules is shown. Selected bond lengths (Å) and angles (°): Cu–Br 2.309(1)‐2.3343(9), Cu–P 2.252(1)‐2.269(1), P(Cu)–P(Cu) 2.198(2)‐2.204(2), P–P 2.171(2)‐2.192(2), P–Cu–Br 117.10(5)‐121.70(5), P–Cu–P 119.62(5)‐121.66(6).

The reaction of *cyclo*‐(P_3_ *t*Bu_3_) with CuBr (1:1) gave the dinuclear complex [(CuBr)_2_{*cyclo*‐(P_3_ *t*Bu_3_)}_2_] (**1**) which was isolated as colorless crystals. Single crystal X‐ray diffraction (SC‐XRD) showed two bis‐monodentate *cyclo*‐(P_3_ *t*Bu_3_) ligands (κ*P* ^1^,κ*P* ^2^ bonding mode) bridging two CuBr moieties which are coordinated in a trigonal‐planar coordination mode thus forming a 6‐membered Cu_2_P_4_ metallacycle (Scheme 2 and Figure 1) with a slightly distorted boat conformation (average P−P−Cu−P torsion angle 48.89(9)°). Remarkably, the *tert*‐butyl substituents of the two coordinating P atoms have a *cis* orientation (mean torsion angle −1.3°) thus presenting the first *cyclo*‐P_3_R_3_ complex with bonding mode **IIb** (Scheme 1) in contrast to **IIa** which has been observed so far for bimetallic complexes of this ligand. Both ligands are oriented in the same way within the metallacycle with the *tert*‐butyl groups of all four coordinating P atoms located on the same side of the metallacycle, resulting in *C*_2_ *_v_* symmetry of the complex and an overall *cis* configuration. Interestingly, the complex crystallizes with three independent molecules in the asymmetric unit (*Z’* = 3) — all of them in general positions — which is rarely observed.^[^ ^26^ ^]^

Further analytical data support the molecular structure. Elemental analysis and mass spectrometry corroborated the composition of complex **1**. In the high‐resolution mass spectrum, an ion peak for [M − Br]^+^ was observed. The characteristic double band caused by the asymmetric deformation vibration of the *t*Bu group was present in the IR spectrum (see Supporting Information). In addition, the ^1^H NMR spectrum (CDCl_3_, Figure S9) showed a pseudo‐doublet and broad singlet with an intensity ratio of about 1:2, similar to the free ligand (1.03 and 1.27 ppm),^[^ ^27^, ^28^ ^]^ but at higher chemical shifts (1.30 and 1.52 ppm) and significantly broadened, as expected for the coordination of the quadrupolar copper nuclei (^63^Cu/^65^Cu, both *I* = ^3^/_2_ with 69% and 31% natural abundance, respectively).

In contrast, the ^31^P{^1^H} NMR data was not consistent. The spectrum showed two multiplets with intensity ratio 1:2, which, similar to the free ligand,^[^ ^27^, ^28^ ^]^ appear as a pseudo‐doublet and pseudo‐triplet. Using full line‐shape analysis, the ^31^P{^1^H} NMR spectrum (Figure S14, Supporting Information) could indeed be refined as an A_2_B spin system with no coupling between the two ligands. However, according to the molecular structure (Figure 1), ^2^ *J*_PP_ coupling constants between the two ligands (via the Cu atoms), which should be comparably large with respect to those within the ligands, should be observed and thus a more complicated AA′BB′B″B^‴^ spin system is expected.

**Figure 2:**
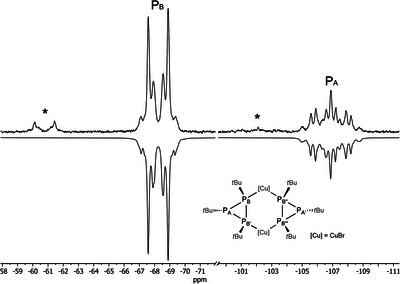
Experimental (top) and simulated (bottom) ^31^P{^1^H} NMR spectrum of complex **1** (*cis* isomer) at −60°C in CDCl_3_. The spectrum was refined as an AA′BB′B″B^‴^ spin system with *C*_2_ *_v_* symmetry (*R* = 0.87%). Selected coupling constants [Hz]: ^1^ *J*_AB_ = −211.4(1), ^1^ *J*_BB’_ = −182(3), ^2^ *J*_BB’’_ = +139.8(4). The asterisk marks the *trans* isomer.

Therefore, complex **1** was investigated with VT‐NMR spectroscopy to clarify if dynamic effects are responsible for this discrepancy. As suspected, the two multiplets become decisively more line‐rich at −60°C, and indeed this NMR spectrum could successfully be refined as an AA′BB′B″B^‴^ spin system by automated line‐shape analysis (Figure 2). The coupling constants thus obtained are in excellent agreement with the solid‐state molecular structure: the ^1^ *J*_PP_ coupling constants (negative, large value) confirm the connectivity within the ligand and, more importantly, the ^2^ *J*_PP_ coupling constants (between P_B_ and P_B’’_, Figure 2) are large and positive which indicates a *cis* relation^[^ ^29^ ^]^ between these P atoms as observed for the overall *cis* configuration of the solid‐state molecular structure.

Furthermore, the ^31^P{^1^H} NMR spectrum at −60°C showed two additional complex multiplets with a low intensity. Accordingly, at low temperature, a second, smaller pair of doublets appeared in the ^1^H NMR spectrum. To rule out the possibility of impurities, a Rietveld refinement of the powder X‐ray diffraction data was performed using the model obtained from the single crystal X‐ray diffraction. The successful refinement (Figure S4, see Supporting Information for details) confirmed that the synthesized material is phase pure and corresponds to the solid‐state structure observed with SC‐XRD. Consequently, we concluded that the additional small signals are caused by a configurational isomer of complex **1** in which the two ligands show a *trans* relationship (**1‐trans**, Scheme 2). Indeed, an analogous complex was observed for silver(I) (complex **4**, vide infra). According to DFT calculations using the r2scan‐3c/def2‐TZVP (ESI), the *cis* isomer **1‐cis** is indeed only +9.1 kJ/mol more stable than the *trans* isomer **1‐trans**, which is in agreement with the observation from VT‐NMR spectroscopy. Apparently, complex **1** undergoes a rapid equilibrium between both isomers at room temperature leading to the simplified ^31^P{^1^H} NMR spectrum (Figure S14, Supporting Information). A dissociative mechanism in which the Cu–P bonds are continuously broken and reformed, can be assumed based on the unexpectedly simple NMR spin system observed at room temperature.

### 2.2 Tetranuclear Copper Complex

**Scheme 3:**
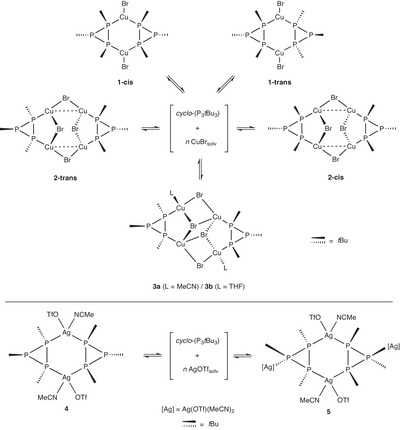
Dynamic relation of copper(I) (top) and silver(I) (bottom) complexes in solution.

**Figure 3:**
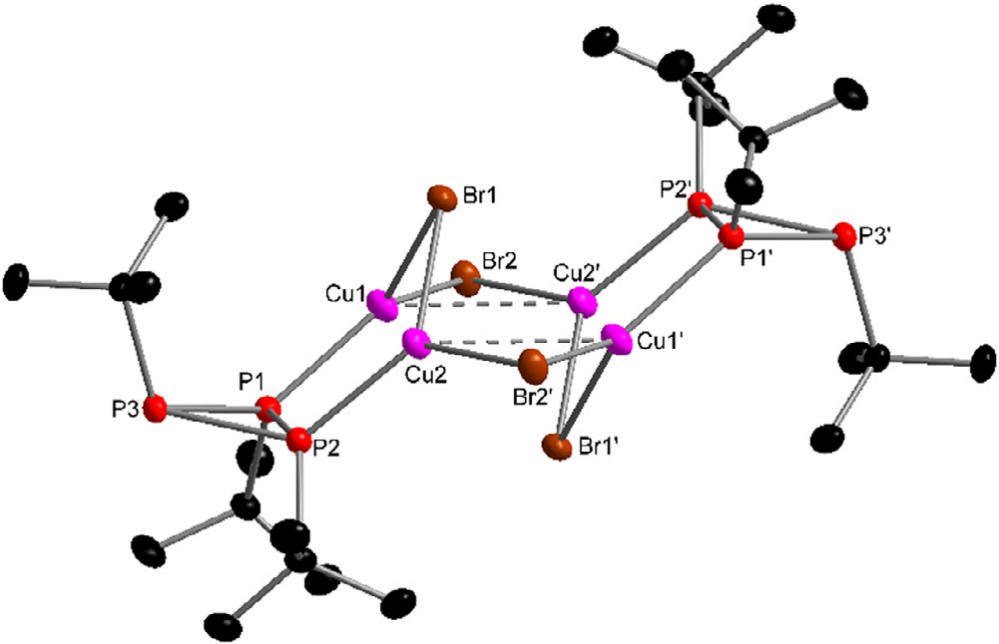
Molecular structure of complex **2**. Selected bond lengths (Å) and angles (°): Cu···Cu 2.8675(5)‐3.0320(5), Cu–Br1 2.4510(5)‐2.4220(4), Cu−Br2 2.3897(5)‐2.4156(4), Cu–P 2.2104(8)‐2.2293(8), Cu1−Br2−Cu2′ 73.27(1), Cu1−Br1−Cu2 76.95(1).

When reacting *cyclo*‐(P_3_ *t*Bu_3_) with [CuBr(SMe_2_)] (1:2), the tetranuclear complex [Cu_4_(μ‐Br)_4_{*cyclo*‐(P_3_ *t*Bu_3_)}_2_] (**2**) was obtained (Scheme 3). The molecular structure was elucidated by SC‐XRD. The complex contains two bis‐monodentate *cyclo*‐triphosphane ligands each coordinating a pair of Cu^I^ atoms in a similar fashion to the dinuclear complex **1**. However, in contrast to **1**, the complex **2** features a Cu_4_Br_4_ core with a planar Cu_4_Br_2_ ring (Figure 3). Complex **2** furthermore resides on a crystallographic inversion center (space group *P*2_1_/*c*, *Z*′ = 0.5). Each of the symmetry‐related Cu atoms is coordinated in an almost perfect trigonal‐planar fashion. An additional close contact between all pairs of Cu atoms (Cu1···Cu2′ 2.8675(5) and Cu1···Cu2 3.0320(5) Å) is observed, indicating a cuprophilic interaction.^[^ ^30^ ^]^ As in complex **1**, the two coordinating P atoms within each ligand have a *cis* relationship (torsion angle −3.59°). In contrast to **1**, the *t*Bu groups of the pairs of coordinating P atoms have a *trans* relationship (inversion center), with an overall *C*_2_ *_h_* symmetry of the molecule. The crystal structure also revealed two non‐classical hydrogen bonds between the bridging bromine atom Br2 and two surrounding methyl groups (Br···(H)C 3.899(3) Å) effectively leading to one‐dimensional chains along the crystallographic [100] direction (Figure S1, Supporting Information).

A CSD search to find similar complexes (CSD reference codes are indicated) yielded similar Cu^I^ halide complexes with κ^2^‐coordinating ligands which mostly differ in the distances of the cuprophilic interactions and the copper‐halide bond lengths of the bridging halogen atoms. Most similar [Cu_4_I_4_R_2_] complexes are FUDLAT^[^ ^31^ ^]^ and CUCDPM^[^ ^32^ ^]^ with similar copper···copper distances ranging from 2.841 to 2.905 Å, whereas UHIFUM,^[^ ^33^ ^]^ DOXCAV,^[^ ^34^ ^]^ and IDALUW^[^ ^35^ ^]^ are examples with even shorter copper···copper distances (2.540 to 2.650 Å). Elemental analysis, mass spectrometry, IR and ^1^H NMR spectroscopy agree with the molecular structure observed in the solid state. The ^1^H NMR spectrum is furthermore similar to complex **1**. A Rietveld refinement of the powder diffraction pattern with the structure model from SC‐XRD confirmed phase purity of the bulk material (Figure S6, Supporting Information). However, the ^31^P{^1^H} NMR spectrum was less conclusive. Two multiplets with pseudo‐doublet and pseudo‐triplet structure were observed, similar to complex **1**. Accordingly, the spectrum could be refined as an A_2_B spin system. The ^1^ *J*_PP_ (−227.2 Hz) obtained from this refinement is slightly larger compared to the free ligand (−201.1 Hz) which is in accordance with the molecular structure of **2** where a shorter bond is observed for the respective phosphorus atoms. In contrast to complex **1**, no significantly large P–P coupling *between* the ligands (^4^ *J*_PP_) is expected, and thus the spectrum as well the refinement as A_2_B spin system is in principle consistent with the molecular structure observed in the solid state. However, the possibility of a dynamic equilibrium as observed for complex **1** cannot be ruled out and is, in consideration of the similarity of both complexes and the strong resemblance of the spectra, very likely. DFT calculations (r2scan‐3c/def2‐TZVP) were used to explore other isomers with similar energies. The calculations showed that the configurational *cis* isomer (**2‐*cis***) is indeed only +3.9 kJ/mol less stable than the *trans* isomer (**2‐*trans***) observed in the solid state. Therefore, we concluded that this complex also undergoes dynamic interconversion reactions in solution.

### 2.3 Solvated Tetranuclear Copper Complex

**Figure 4:**
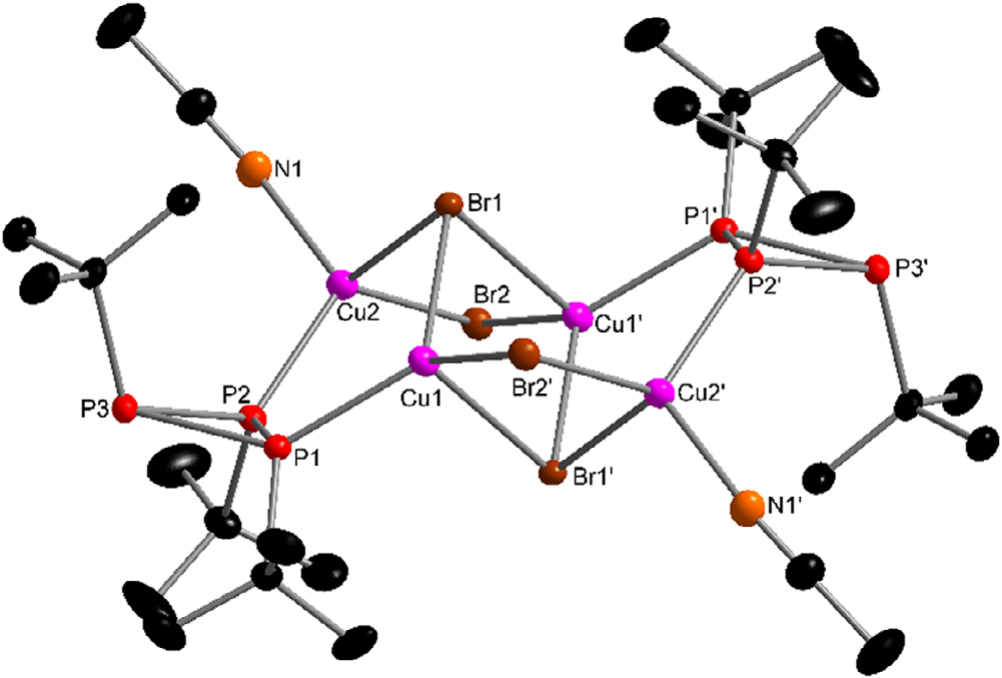
Molecular structure of **3a**. Selected bond lengths (Å) and angles (°): Cu1–Br1 2.4915(3), Cu1–Br1’ 2.7051(3), Cu1–Br2’ 2.4298(4), Cu2–Br1 2.5690(4), Cu2–Br2 2.4950(3), Cu2–N1 2.028(2), P1–Cu1–Br1 116.63(2), P1–Cu1–Br2’ 126.65(2), Br1–Cu1–Br2’ 111.34(1), Br1–Cu1–Br1’ 92.91(1), P1–Cu1–Br1’ 100.46(1), Br1’–Cu1–Br2 99.04(1), P2–Cu2–Br1 109.84(1), P2–Cu2–Br2 116.93(1), P2–Cu2–N1 113.68(5), Br1–Cu2–Br2 101.07(1), Br1–Cu2–N1 105.07(5), N1–Cu2–Br2 108.89(5).

During the preparation of the tetranuclear complex **2** from CuBr instead of [CuBr(SMe_2_)], two further complexes were isolated as colorless single crystals and characterized by SC‐XRD. The complexes were crystallized from acetonitrile or THF and contain the respective solvent molecules as additional co‐ligands. The molecular structure of the complex [Cu_4_(μ‐Br)_4_{*cyclo*‐(P_3_ *t*Bu_3_)}_2_(MeCN)_2_] (**3a**) is displayed in Figure 4. The centrosymmetric complex can formally be derived from complex **2** by the addition of MeCN co‐ligands to two of the four Cu atoms located diametrically opposite to each other. These atoms (Cu2 and Cu2′) thus change their coordination environment from trigonal planar to distorted tetrahedral (*τ*_4_’^[^ ^36^ ^]^ = 0.8; 1 for perfect tetrahedral and 0 for perfect square‐planar). Furthermore, two bromido ligands are now bridging three copper cations (μ_3_ bonding mode) resulting in a tetrahedral coordination environment for Cu1 and Cu1′ (*τ*_4_’ = 0.91). Furthermore, a parallel shift of the two {Cu_2_(*cyclo*‐P_3_ *t*Bu_3_)} units leads to a reduction of symmetry from *C*_2_ *_h_* to *C_i_*. A similar arrangement of copper and donor atoms was already observed in other complexes of the type Cu_4_X_4_L_4_ (L donor atoms) and can be described as stepped or face‐shared dicubane.^[^ ^37^ ^]^ The bond lengths and angles in **3a** are in the expected ranges (Figure 4).^[^ ^38^, ^39^ ^]^ Interestingly, the bond between the coordinating P atoms with the *tert*‐butyl groups in *cis* position (P1–P2, 2.196(1) Å) is slightly shorter compared to the free phosphane (2.213–2.218 Å)^[^ ^27^, ^28^ ^]^ which is probably caused by the coordination of the two Cu atoms.

All attempts to confirm the molecular structure of complex **3a** with other methods than SC‐XRD were unsuccessful. The ^1^H NMR spectrum (in CDCl_3_) showed a doublet and a triplet which can be assigned to the *t*Bu groups of the ligand. An additional signal is observed at 2.01 ppm which can be attributed to MeCN. This chemical shift, however, matches very well with the solvent residual signal of MeCN in CDCl_3_.^[^ ^40^ ^]^ Likewise, the signal at 116.3 ppm in the ^13^C{^1^H} NMR spectrum indicates that the copper–acetonitrile bond is labile and dissociates in solution. To verify this presumption aliquots of MeCN were added in an NMR titration experiment. No additional signal, but a mere increase of the signal intensity at 2.01 ppm for free MeCN, was observed (Table S3, Supporting Information). These findings are further corroborated by the ^31^P{^1^H} NMR spectrum which is identical with the solvent‐free complex **2**. Elemental analysis performed on different samples of complex **3a** showed compositions which are in line with a decreasing amount of coordinated MeCN, namely *n* = 1.8 and 0.8 in [Cu_4_(μ‐Br)_4_(MeCN)_*n*_{*cyclo*‐(P_3_ *t*Bu_3_)}_2_]), but not the expected value for *n* = 2. A PXRD analysis, carried out to ascertain whether the bulk material corresponds to the single crystal solid‐state structure of the complex with (**3a**) or without (**2**) acetonitrile, was inconclusive (Figure S8, Supporting Information). Finally, FTIR spectroscopy did not show the characteristic C≡N stretching vibration for the MeCN ligand. Therefore, we conclude that the MeCN ligands in complex **3a** are only very weakly bound and readily lost in solution as well as in the solid material.

In addition to complex **3a** with acetonitrile, a second complex containing coordinated THF was obtained as colorless single crystals with the overall formula [Cu_4_(μ‐Br)_4_{*cyclo*‐(P_3_ *t*Bu_3_)}_2_(THF)_2−_ *_n_*]·*n*THF (*n* = 0.52) (**3b**). The crystal structure (Figure S2, Supporting Information) contains an interesting positional disorder. The complex is located on an inversion center (space group *Pbca*) with only half of the molecule being symmetry‐independent (*Z*′ = 0.5). Herein, two split positions could be refined for one of the independent copper atoms: copper atom Cu2A (48%) is coordinated by a THF molecule and has a distorted tetrahedral coordination environment, very similar to the copper atom coordinated by acetonitrile in complex **3a**. Additionally, in very close proximity to Cu2A, a copper atom (Cu2B, 52%) with trigonal‐planar coordination environment, as was observed in complex **2**, is located. In this case, the THF molecule is not coordinated at the copper atom but is instead included as non‐coordinating molecule in the crystal structure in close proximity to the complex. This disorder in combination with the location on an inversion center leads to the presence of three distinct complexes in the crystal structure, namely [Cu_4_Br_4_{*cyclo*‐(P_3_ *t*Bu_3_)}_2_]·2THF (THF solvate of **2**, 27%), [Cu_4_Br_4_{*cyclo*‐(P_3_ *t*Bu_3_)}_2_(THF)_2_] (analogous to **3a**, 23%), and [Cu_4_Br_4_{*cyclo*‐(P_3_ *t*Bu_3_)}_2_(THF)]·THF (mixed type of the former two, with 50% abundance). Structural parameters of the three molecules are similar to complexes **2** and **3a**.

The NMR spectra of complexes **3a** and **3b** are identical to complex **2**, suggesting that complexes **3a** and **3b** lose their coordinated solvent molecules in solution. In order to support this hypothesis, we carried out DFT calculations (BP86/def2‐TZVP, Supporting Information). Indeed, the reaction of complex **2** with one or two acetonitrile molecules is strongly endergonic with a reaction energy of +58.2 kJ/mol, suggesting that complexes which contain additional coordinated solvent molecules are stabilized by intermolecular interactions in the solid state.

### 2.4 Dinuclear Silver Complex

**Figure 5:**
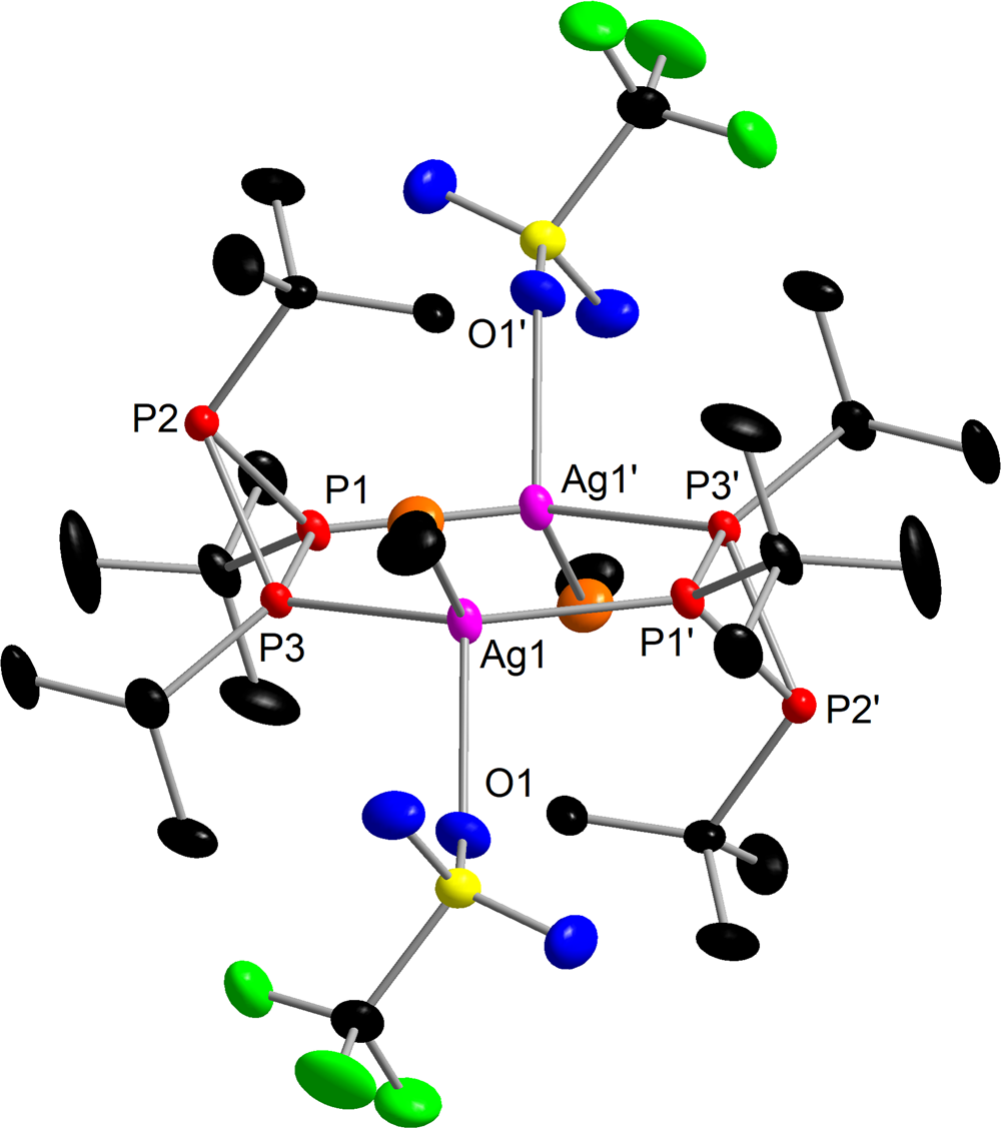
Molecular structure of **4**. Selected bond lengths (Å) and angles (°): Ag−P 2.4600(4)‐2.4606(4), P−P 2.1766(6)‐2.1999(6), Ag1−O1 2.481(1), Ag1−N1 2.368(2), P1−Ag1−P3’ 135.45(2).

The phosphane *cyclo*‐(P_3_ *t*Bu_3_) reacts with AgOTf in acetonitrile to yield the dinuclear complex [{Ag(OTf)(MeCN)}_2_{*cyclo*‐(P_3_ *t*Bu_3_)}_2_] (**4**) as colorless single crystals. X‐ray diffraction analysis revealed a compound with a similar bonding mode of the *cyclo*‐(P_3_ *t*Bu_3_) ligand as observed for the dinuclear copper complex **1**: two bis‐monodentate phosphane ligands bridge (in κ*P* ^1^,κ*P* ^2^ bonding mode) two silver atoms forming an almost perfectly planar Ag_2_P_4_ metallacycle (average distance from best plane 0.031 Å). The silver(I) atoms feature a distorted tetrahedral coordination environment which is most evident in the P–Ag–P bond angle with 135.45(2)° (Figure 5). In contrast to complex **1**, the configuration of the *tert*‐butyl groups with respect to each other (i.e., between the ligands) is *trans*. The overall *C_i_* symmetry is represented in the crystal structure by the presence of a crystallographic inversion center which is located in the center of the six‐membered Ag_2_P_4_ metallacycle. Parameters of the molecular structure are within expected ranges (Figure 5). As for complex **1**, elemental analysis, mass spectrometry, IR and ^1^H NMR spectroscopy support the findings from SC‐XRD, again, with the exception of the ^31^P{^1^H} NMR spectrum.

As for **1**, a pseudo‐doublet (P_A_) and pseudo‐triplet (P_B_) were observed in the ^31^P{^1^H} NMR spectrum. Interestingly, the signal for P_B_ (representing P1/P3 which coordinate Ag1) is slightly shifted upfield compared to the free ligand, meaning it is shielded upon coordination, as has also been observed for other silver(I) phosphane complexes.^[^ ^40^ ^]^ The apparent A_2_B spin system is too simple for the molecular structure, as also here significant ^2^ *J*_PP_ coupling should lead to a more complicated spin system. Furthermore, the spin system expected for complex **4** is considerably more complicated compared to complex **1** due to the presence of the two silver isotopes (^107^Ag/^109^Ag both *I* = ^1^/_2_ with 52% and 48% abundance, respectively)^[^ ^41^ ^]^ which leads to three NMR‐distinguishable complexes: two with an AA′A″A^‴^BB′XX′ spin system for the homo‐isotopic complexes (**4**‐^107^Ag_2_ and **4**‐^109^Ag_2_) and one with an AA′BB′CC′MX′ spin system for **4**‐^107^Ag,^109^Ag. As the gyromagnetic ratio of the two isotopes, which determines the ratio between the coupling constants, is only 1.15 in case of silver (*J*_109AgX_/*J*_107AgX_), a severe overlap of these three spin systems can be expected. Indeed, when cooling the sample to −60°C the two signals clearly change appearance to a higher‐order NMR spectrum. However, all attempts to refine the three spin systems failed. Silver isotope enrichment was demonstrated to enable full line‐shape analysis of the higher‐order NMR spectrum of the phosphane complexes [Ag_2_(R_2_PCH_2_PR_2_)_3_](AsF_6_) (R = Me, Ph)^[^ ^42^ ^]^ and thus represents a possible solution for future work.

Interestingly, in contrast to complex **1** no additional small signals indicating the presence of a second isomer were observed in the ^31^P{^1^H} NMR spectrum at −60°C. DFT calculations (BP86/def2‐TZVP) conducted to evaluate the possibility for formation of further isomers showed that the *trans* isomer is 60 kJ/mol more stable than the *cis* isomer. Presumably, the different coordination environment with an additional co‐ligand and the planarity of the metallacycle compared to the copper complex **1** render the *cis* configuration in **4** much more unstable. This is in accordance with the solid state where the *trans* isomer of **4** is observed. Furthermore, this energy barrier is in fact too high to be overcome at −60°C which explains why the other isomer is not observed.

However, while no cis/trans interconversion is expected for complex **4**, a broad signal is observed in the ^31^P{^1^H} NMR spectrum at room temperature which unequivocally points to a dynamic behavior. We conclude that a continuous dissociation and reformation of the metal complex **4** in solution similar to the mechanism discussed for complex **1** takes place.

### 2.5 Tetranuclear Silver Complex

While optimizing the synthesis of complex **4**, colorless single crystals of another compound could be isolated. The SC‐XRD analysis showed the formation of the tetranuclear complex **5**, which can formally be derived from complex **4** by addition of {Ag(OTf)(MeCN)_2_} units to the non‐coordinating phosphorus atoms in the *cyclo*‐triphosphane ligand. Complex **5** crystallizes (Figure S3) as a centrosymmetric compound (space group *P*2_1_/*c*, *Z*′ = 0.5). The Ag2−P3 bond of the additional silver atom (2.437(1) Å) is slightly longer than the Ag−P bonds in the Ag_2_P_4_ metallacycle (*d*(Ag−P) = 2.470(1)–2.478(1) Å). The remaining structural parameters are very similar to complex **4**.

IR spectroscopy and elemental analysis of crystals of **5** confirmed the observations made by X‐ray diffraction. Surprisingly, ^1^H and ^31^P{^1^H} NMR spectra of crystals of complex **5** were identical with the NMR spectra of the dinuclear complex **4**. The ^31^P{^1^H} NMR spectrum at room temperature showed the same pseudo‐doublet and pseudo‐triplet. However, for a tetranuclear complex an additional splitting of the pseudo‐doublet corresponding to the P atom that coordinates the {Ag(OTf)(MeCN)_2_} unit is expected as ^1^ *J*_AgP_ couplings are in the range of 300–400 Hz^[^ ^41^ ^]^ and should thus have a clear effect at least on this multiplet. These results demonstrate that the species present in the solution is not consistent with the molecular structure observed for complex **5** in the solid state and that the latter is prone to similar dynamic interconversion reactions as observed for complexes **1‐4** in solution involving continuous dissociation and reformation of the M−P bonds.

It has to be noted that it was not possible to reproduce the formation of complex **5**. The original sample was obtained from a reaction of *cyclo*‐(P_3_ *t*Bu_3_) with two equivalents of AgOTf with MeCN as solvent. Recreating these conditions, use of a different silver(I) precursors such as AgCl, or even reacting complex **4** with one equivalent as well as excesses of AgOTf did not lead to a successful reproduction of complex **5**. Analysis with single crystal or powder X‐ray diffraction showed that in each case the obtained materials consisted of complex **4** and the silver(I) precursor. It is therefore concluded that the energy gain for the coordination of the third phosphorus atom in the ligand to give complex **5** must be very small. This is in accordance with observations made for other multinuclear complexes containing oligophosphanes of type P*_x_* R*_y_* where usually an increasing number of coordinated metal atoms leads to decrease of the coordinating power of the phosphane.^[^ ^2^, ^42^ ^]^ Due to the assumed low reaction energy, the lattice energy and the general tendency to crystallize depending on the respective conditions must therefore be the determining factors for the outcome of the reaction. These clearly seem to be more pronounced for the formation of complex **5**.

## 3 Conclusion

This work reports the synthesis of di‐ and tetranuclear copper(I) and silver(I) complexes each containing two *cyclo*‐(P_3_ *t*Bu_3_) ligands. The dinuclear copper complex **1**, the tetranuclear copper complex **2**, and the dinuclear silver complex **4** were characterized by several methods which corroborated their respective molecular solid‐state structures, while NMR spectroscopic studies shed light on their behavior in solution.

In solution, the dinuclear copper complex **1** undergoes an interconversion reaction to a second isomer (**1‐trans**, Scheme 3) as revealed by a second signal set in the ^31^P{^1^H} NMR spectrum at −60°C. The spectrum at room temperature instead shows a single set of broad signals which indicates that this reaction is rather rapid under these conditions. Similarly, the dinuclear silver complex **4** exhibits a fine structure in the ^31^P{^1^H} NMR spectrum at low temperature and one single set of broad signals at room temperature which strongly suggests a similar dynamic behavior. The fact that in contrast to **1** no second isomer is observed in the case of **4** discounts the possibility of an *intra*molecular process and instead strongly suggests, for both complexes **1** and **4**, a dissociation in solution to afford an equilibrium between different compounds, including a continuous cleavage and reforming of the M−P bonds. Similar cases where this is evident are known: the gold(I) complex [(AuCl)_2_{*cyclo*‐(P_4_ *t*Bu_4_}] forms an equilibrium in solution between the 1,2‐ and the 1,3‐isomer where the structure of the ligand excludes an intramolecular interconversion reaction.^[^ ^14^ ^]^ Furthermore, labile M−P bonds are known for silver(I) complexes of bis‐phosphanes. Thus, at room temperature [Ag_2_(dppm)_3_] (dppm = PPh_2_CH_2_PPh_2_) undergoes a rapid intramolecular conversion during which the metal–phosphorus bond is continuously broken and reformed.^[^ ^43^ ^]^

The presence of multiple equilibria as visualized in Scheme 3 between various species is further corroborated by the fact that the NMR spectra of the di‐ and tetranuclear complexes are very similar (in case of copper, **1** and **2**) or identical (in case of silver, **4** and **5**). For each system, it is thus likely that both complexes (**1** and **2** or **4** and **5**) form in solution as soon as the metal salt and the *cyclo*‐(P_3_ *t*Bu_3_) ligand are present with their respective fractions depending only on the metal/ligand ratio. Accordingly, only the chemical shifts of the signals in the ^31^P{^1^H} NMR spectrum are a reflection of the phosphorus atoms’ chemical environment averaged over time, whereas the ^1^ *J*_PP_ coupling, solely determined by the invariant ligand structure, remains unchanged. The difference between both transition metals can be explained by their coordination‐induced shift on ^31^P nuclei. For silver(I) this is known to be very small which causes an indiscernible difference between the spectra of the di‐ and tetranuclear complex.

These equilibria also explain why metal complexes with a different metal/ligand ratio as present in the reaction mixture can be isolated. Similar observations have been made by Scherer et al. who isolated the complex [Cu_2_(μ‐Cl)_2_(THF){*cyclo*‐(P_5_Ph_5_)}_2_] from the 2:1 reaction (CuCl:L) and the complex [Cu_4_(μ‐Cl)_4_{*cyclo*‐(P_5_Ph_5_)}_2_] from the 1:2 reaction.^[^ ^44^ ^]^

In addition to the di‐ and tetranuclear complex, further complexes form in solution once metal salt and *cyclo*‐(P_3_ *t*Bu_3_) ligand are present. In case of the system copper(I)/*cyclo*‐(P_3_ *t*Bu_3_) the elusive solvated, highly labile complexes **3a** and **3b** could be identified in addition to complexes **1‐cis**, **1‐trans** and **2**. The lability of the latter becomes apparent in the crystal structure of the complex with THF co‐ligands (**3b**) which co‐crystallized with the simply clathrated complex. Further short‐lived species which have evaded detection are entirely conceivable, also in the case of silver(I).

Our results suggest that these equilibria could also be relevant for other complexes, but might have been overlooked if NMR spectroscopy did not indicate any dynamic behavior.

## Supporting Information

Supporting Information gives details about the syntheses and characterization of complexes **1, 2**, **3a**, **3b**, **4** and **5**, X‐ray and powder X‐ray diffraction, and quantum chemical calculations. The authors have cited additional references within the Supporting Information.^[45–63]^ Supporting Information also includes a short summary of the thermal analysis of complex **2**.

## Conflict of Interests

The authors declare no conflict of interest.

## Supporting information



Supporting Information
