# Supplementary material for: Dynamic Copper(I) and Silver(I) Complexes of Tri‐tert‐butyl‐cyclotriphosphane
Source: Chemistry. 2025 Apr 17;31(30):e202500746. doi: 10.1002/chem.202500746 (PMC12117173; doi:10.1002/chem.202500746)
Supplement: Supplementary file 1 — Supporting Information [file CHEM-31-e202500746-s001.pdf]

Electronic Supporting Information:

Dynamic Copper(I) and Silver(I) Complexes of Tri-*tert*-butyl-cyclotriphosphane

Toni Grell,*[a] Peter Wonneberger,[b] Divine Mbom Yufanyi,[b,c] Peter Lönnecke,[b] Evamarie Hey-Hawkins*[b,d,e]

In Memory of Professor Ian Manners – an exceptional scientist and a wonderful colleague

[a] Dr. T. Grell
Dipartimento di Chimica
Università degli Studi di Milano
Via Camillo Golgi 19, 20131 Milan, Italy
E-mail: toni.grell89@gmail.com

[b] Dr. P. Wonneberger, Dr. P. Lönnecke, Prof. Dr. h.c. mult. E. Hey-Hawkins
Faculty of Chemistry and Mineralogy
Leipzig University
Johannisallee 29, 04103 Leipzig, Germany
E-mail: hey@uni-leipzig.de

[c] Dr. D. M. Yufanyi
Department of Fundamental Science, Higher Technical Teacher Training College Bambili
The University of Bamenda
P.O. Box 39 Bambili, Bamenda, Cameroon

[d] Prof. Dr. h.c. mult. E. Hey-Hawkins
New address: Faculty of Chemistry and Mineralogy, Institute of Bioanalytical Chemistry, Leipzig University, Deutscher Platz 5, 04103 Leipzig, Germany

[e] Faculty of Chemistry and Chemical Engineering, Department of Chemistry, Babes-Bolyai University, 1, Kogalniceanu str., RO-400084 Cluj-Napoca, Romania

Index

1. Single Crystal X-Ray Diffraction (SC-XRD) S2
2. Powder X-Ray Diffraction (PXRD) S4
3. Experimental Section S7
   3.1. General Remarks S7
   3.2. Synthesis of Complex **1** S8
   3.1. Synthesis of Complex **2** S9
   3.2. Synthesis of Complex **3a** S9
   3.3. Synthesis of Complex **3b** S10
   3.1. Synthesis of Complex **4** S11
   3.2. Synthesis of Complex **5** S12
4. Quantum Chemical Calculations S12
5. NMR Measurements and Spectra of the Compounds S12
6. Thermal Analysis S23
7. References S24

1. Single Crystal X-Ray Diffraction (SC-XRD)

Single crystal X-ray data were collected with a GEMINI CCD diffractometer (Rigaku Inc.), λ(Mo-Kα) = 0.71073 Å, T = 130(2) K, empirical absorption corrections with SCALE3 ABSPACK using CrysAlis Pro.[45] All structures were solved by dual space methods using SHELXT-2017.[46] Structure refinement was done with SHELXL-2018[47] by using full-matrix least-square routines against F^2 with the WinGX program suite.[48] All hydrogen atoms were calculated on idealized positions. The figures were generated with the program DIAMOND.[49] In all graphics, hydrogen atoms, disorders, and solvent molecules were omitted for clarity. Thermal ellipsoids are shown with 50% probability. CCDC numbers given in Table S1 contain the supplementary crystallographic data for this paper. These data can be obtained free of charge via https://www.ccdc.cam.ac.uk/structures/ (or from the Cambridge Crystallographic Data Centre, 12 Union Road, Cambridge CB2 1EZ, UK; fax: (+44)1223-336-033; or deposit@ccdc.cam.uk).

Table S1. Summary of crystallographic data for complexes **1**-**5**.

Compound	**1**	**2**	**3a**	**3b**	**4**	**5**
Formula	$C_{24}H_{54}Br_2Cu_2P_6$	$C_{24}H_{54}Br_4Cu_4P_6$	$C_{28}H_{60}Br_4Cu_4N_2P_6$	$C_{32}H_{70}Br_4Cu_4O_2P_6$	$C_{30}H_{60}Ag_2F_6N_2O_6P_6S_2$	$C_{40}H_{72}Ag_4F_{12}N_6O_{12}P_6S_4$
Formula weight	815.39	1102.29	1184.40	1246.50	1124.48	1802.57
Crystal system	Monoclinic	Monoclinic	Triclinic	Orthorhombic	Monoclinic	Monoclinic
Space group	$P2_1/c$	$P2_1/c$	$P\bar{1}$	*Pbca*	$P2_1/n$	$P2_1/c$
a [Å]	16.7022(3)	9.3986(4)	9.8970(6)	18.3677(8)	9.35480(10)	9.9577(3)
b [Å]	28.7363(5)	15.9740(5)	10.7214(5)	13.3592(13)	21.6668(2)	23.0184(8)
c [Å]	22.7006(3)	13.5731(5)	11.8177(6)	19.462(5)	11.74720(10)	14.8286(5)
α [°]	90	90	106.757(4)	90	90	90
β [°]	89.975(2)	107.727(4)	91.101(4)	90	99.3640(10)	99.934(3)
γ [°]	90	90	105.655(4)	90	90	90
V [Å^3]	10895.4(3)	1941.02(13)	1149.76(11)	4775.7(14)	2349.30(4)	3347.91(19)
Z	12	2	1	4	2	2
$\rho_{(calcd)}$ [g·cm^{-3}]	1.491	1.886	1.711	1.734	1.590	1.788
μ [mm^{-1}]	3.649	6.542	5.530	5.332	1.189	1.509
F(000)	4992	1088	588	2496	1144	1800
Cryst. size [mm^3]	0.25 · 0.18 · 0.17	0.08 · 0.08 · 0.05	0.15 · 0.15 · 0.08	0.15 · 0.15 · 0.10	0.38 · 0.17 · 0.10	0.20 · 0.08 · 0.08
θ range [°]	2.283 - 30.645	2.275 - 32.455	2.298 - 32.395	2.218 - 32.648	2.398 - 32.585	2.257 - 32.520
Limiting indices	-23 ≤ h ≤ 23,<br>-40 ≤ k ≤ 39,<br>-32 ≤ l ≤ 32	-13 ≤ h ≤ 14,<br>-23 ≤ k ≤ 23,<br>-19 ≤ l ≤ 18	-14 ≤ h ≤ 14,<br>-15 ≤ k ≤ 15,<br>-17 ≤ l ≤ 17	-19 ≤ h ≤ 26,<br>-15 ≤ k ≤ 20,<br>-29 ≤ l ≤ 25	-13 ≤ h ≤ 13,<br>-32 ≤ k ≤ 32,<br>-16 ≤ l ≤ 16	-14 ≤ h ≤ 14,<br>-34 ≤ k ≤ 34,<br>-22 ≤ l ≤ 21
Reflections collected [$R_{(int)}$]	105894 [0.0745]	24222 [0.0476]	14418 [0.0204]	21869 [0.0472]	68757 [0.0406]	23062 [0.0450]
Data / restraints / parameters	30350 / 0 / 974	6470 / 0 / 181	7479 / 0 / 209	7900 / 0 / 281	8098 / 0 / 254	10873 / 0 / 401
Completeness [%] to θ [°]	99.9 (28.29)	100.0 (25.242)	99.9 (25.242)	100.0 (25.242)	100 (30.51)	99.8 (25.242)
T_{Min} / T_{Max}	1 / 0.96398	1 / 0.92188	1 / 0.75506	1 / 0.92272	1 / 0.93592	1 / 0.80786
R1, wR2 (I > 2σ(I))	0.0517, 0.0822	0.0356, 0.0575	0.0232, 0.0488	0.0431, 0.0747	0.0280, 0.0583	0.0705, 0.1174
R1, wR2 (all data)	0.1023, 0.0981	0.0573, 0.0632	0.0294, 0.0510	0.0791, 0.0877	0.0341, 0.0608	0.1053, 0.1291
Goof [on F^2]	1.020	1.046	1.042	1.069	1.077	1.179
Residual electron density [e·Å^{-3}]	1.152 / -1.152	1.280 / -0.764	0.554 / -0.564	0.933 / -0.751	0.634 / -0.402	1.164 / -1.222
Comments	†1	-	-	-	-	-
CCDC No.	2424883	2424640	2424638	2424641	2424884	2424639

†1: Two-component twin. Twin domain ratio: 0.5223(5) / 0.4777(5). Twin law by rows: -1 0 0 0 -1 0 0 0 1

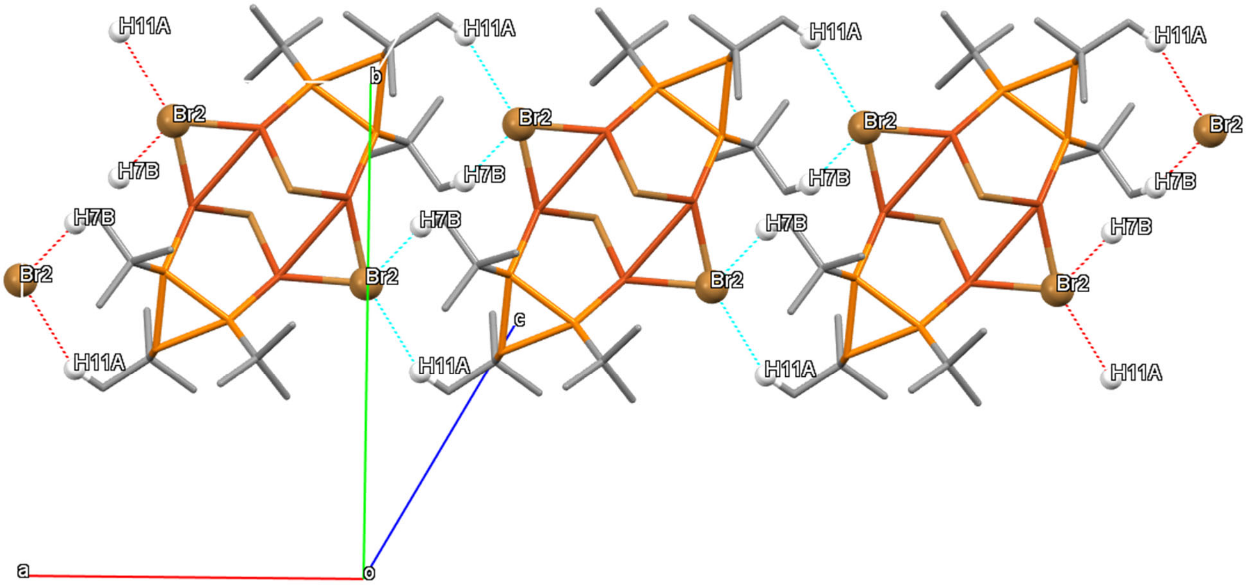

Figure S1. Highlighted interatomic interactions in the crystal structure of complex **2**.

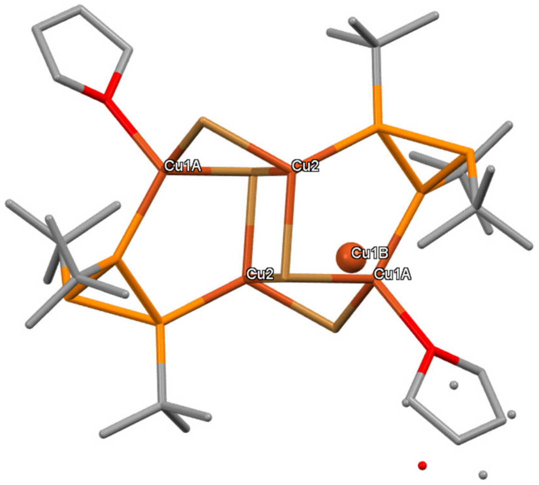

Figure S2. Molecular structure of complex **3b**. The molecule is located on an inversion center. The second split position of the copper atom is only shown for one half of the molecule.

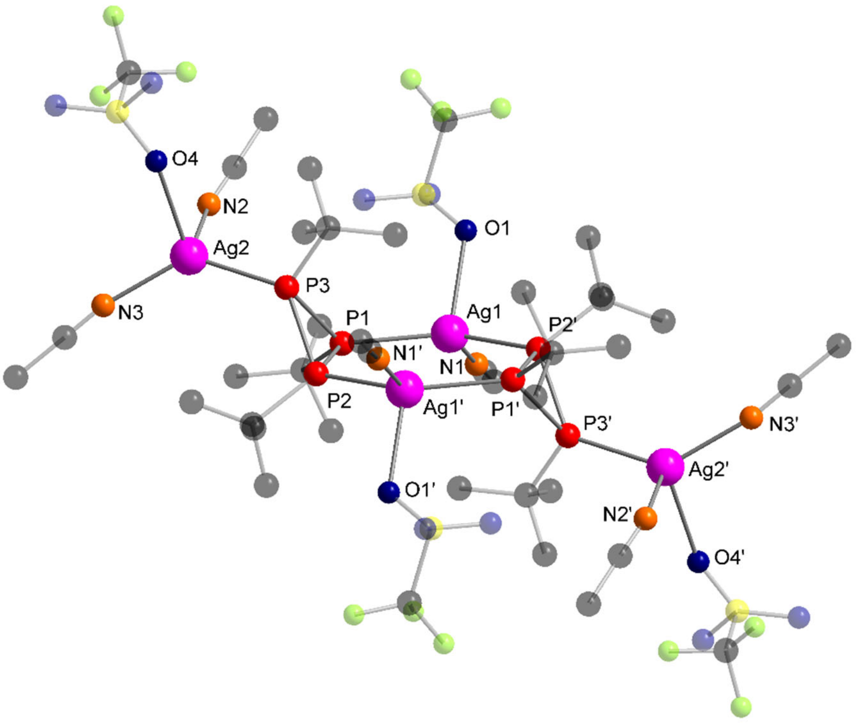

Figure S3. Molecular structure of complex **5**.

2. Powder X-Ray Diffraction (PXRD)

Powder X-ray diffraction data were collected at room temperature on a STADI-P diffractometer (STOE) with a silicon solid-state detector Methyn-1K (DECTRIS), a germanium single crystal monochromator, and a copper anode (Cu-Kα, λ = 1.540598 Å) as radiation source. Samples were measured in sealed glass capillaries (inner diameter 0.7 mm, Hilgenberg) with Debye-Scherrer geometry. Processing of the raw data was carried out with the diffractometer software WinXPow (STOE).[50]

Rietveld refinements were carried out by using a fundamental parameter approach and the software TOPAS-Academic V6 (BRUKER).[51] The background was modelled by a polynomial function (6th order) of the Chebyshev type. Peak shapes were described by the fundamental parameter approach. Separate isotropic thermal parameters were assigned and allowed to freely refine for bromine, copper and all remaining elements. A Le Bail refinement was used in order to obtain initial cell parameters based on the ones of the respective single crystal structure. Fractional coordinates of the single crystal structures were used for the Rietveld refinement treating the complex molecules as rigid bodies for which independent translation and rotation was allowed. The final structures after refinement were checked (cell parameters, molecular parameters, and packing, Tables S2) to confirm their agreement with the single crystal structure parameters. Besides the Rietveld refinements the PXRD pattern was also compared visually with the pattern simulated with the single crystal structure model using MERCURY[52] (Figs. S4-S8).

Table S2. Summary of the Rietveld refinement of the solid-state structures of $[(CuBr)_2\{$*cyclo*$\text{-}(P_3tBu_3)\}_2]$ (**1**) and $[(CuBr)_4\{$*cyclo*$\text{-}(P_3tBu_3)\}_2]$ (**2**).

Compound	$[(CuBr)_2\{$*cyclo*$\text{-}(P_3tBu_3)\}_2]$ (**1**)	$[(CuBr)_4\{$*cyclo*$\text{-}(P_3tBu_3)\}_2]$ (**2**)
Crystal system	Monoclinic	Monoclinic
Space group	$P2_1/c$	$P2_1/c$
a [Å]	16.82461	9.53950
b [Å]	29.26403	16.29225
c [Å]	23.08990	13.63301
α [°]	90	90
β [°]	90.03186	107.34623
γ [°]	90	90
V [$Å^3$]	11368.4409	2022.47825
Z	12	2
Radiation type	Cu-Kα	Cu-Kα
Temp. [K]	298	298
$2\Theta_{min}$ / $2\Theta_{max}$ [deg]	2-62	2-62
Goodness-of-fit	6.518	7.591
R_{Bragg} [%]	16.812	17.868
R_{exp} [%]	3.250	3.722
R_{wp} [%]	21.186	28.252
R_p [%]	16.767	22.216

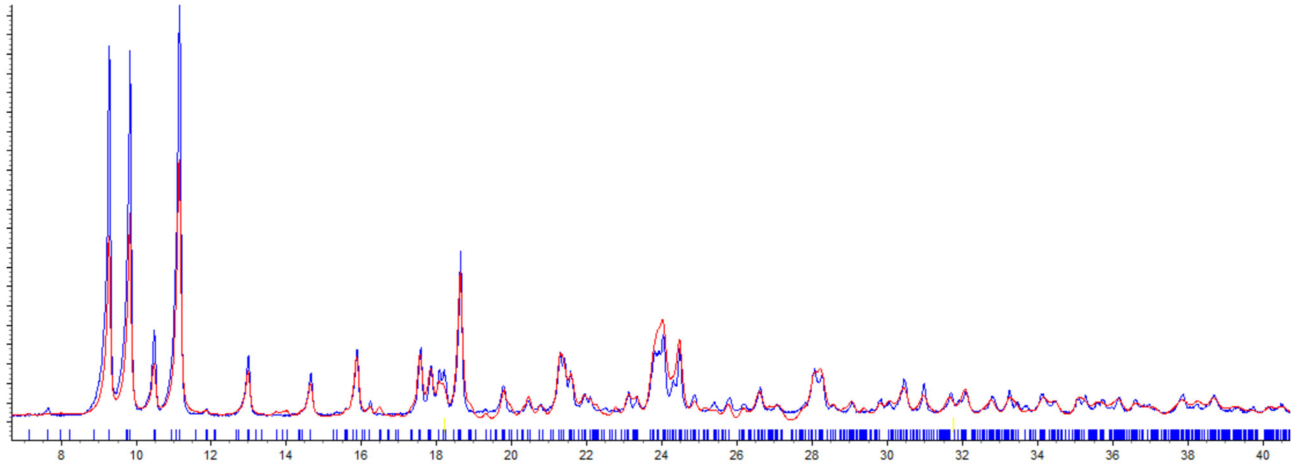

Figure S4. Graphical representation of the Rietveld refinement carried out on the PXRD data of $[(CuBr)_2\{$*cyclo*$\text{-}(P_3tBu_3)\}_2]$ (**1**) with experimental (blue) and calculated (red) pattern. The positions of the Bragg reflections are indicated by blue ticks. Abscissa 2Θ in °.

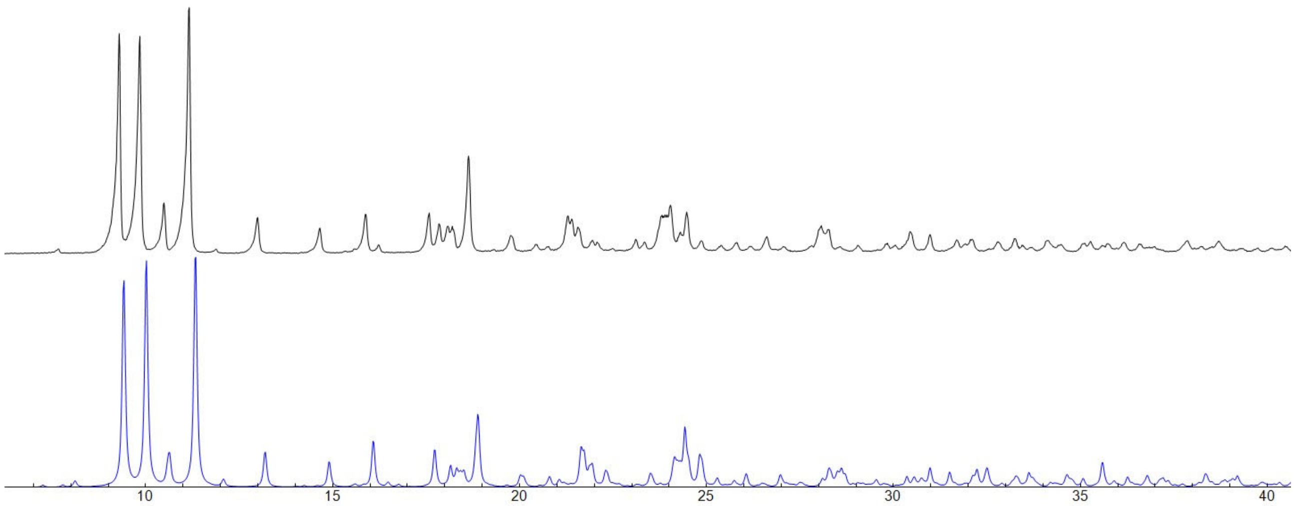

Figure S5. Visual comparison of the experimental powder pattern of [$(CuBr)_2${*cyclo*-$(P_3tBu_3)\}_2$] (**1**) (black, top) and the pattern simulated from the single crystal structure model recorded at 130 K (blue, bottom). Abscissa 2Θ in °.

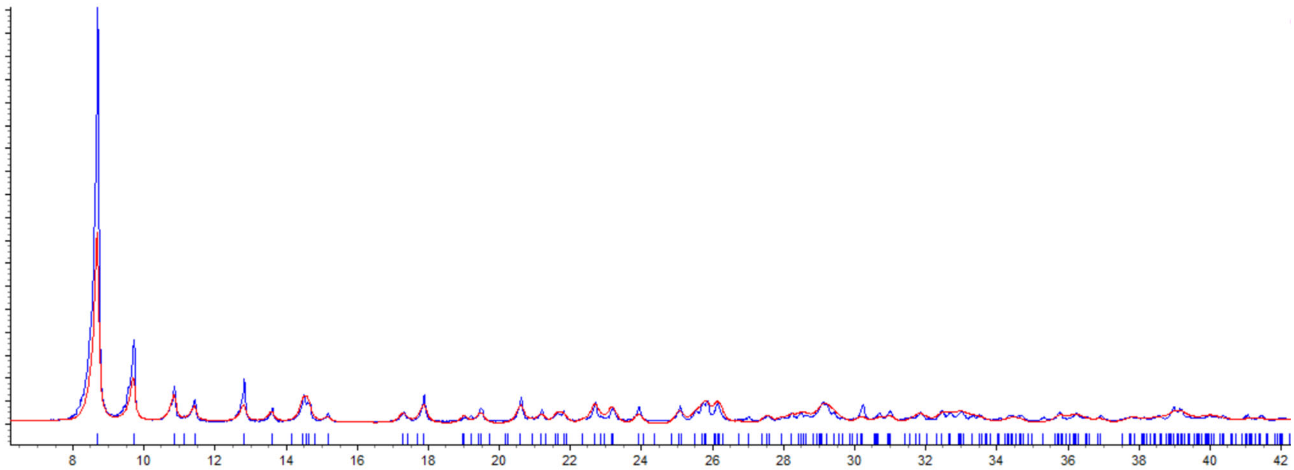

Figure S6. Graphical representation of the Rietveld refinement carried out on the PXRD data of [$(CuBr)_4${*cyclo*-$(P_3tBu_3)\}_2$] (**2**) with experimental (blue) and calculated (red) pattern. The positions of the Bragg reflections are indicated by blue ticks. Abscissa 2Θ in °.

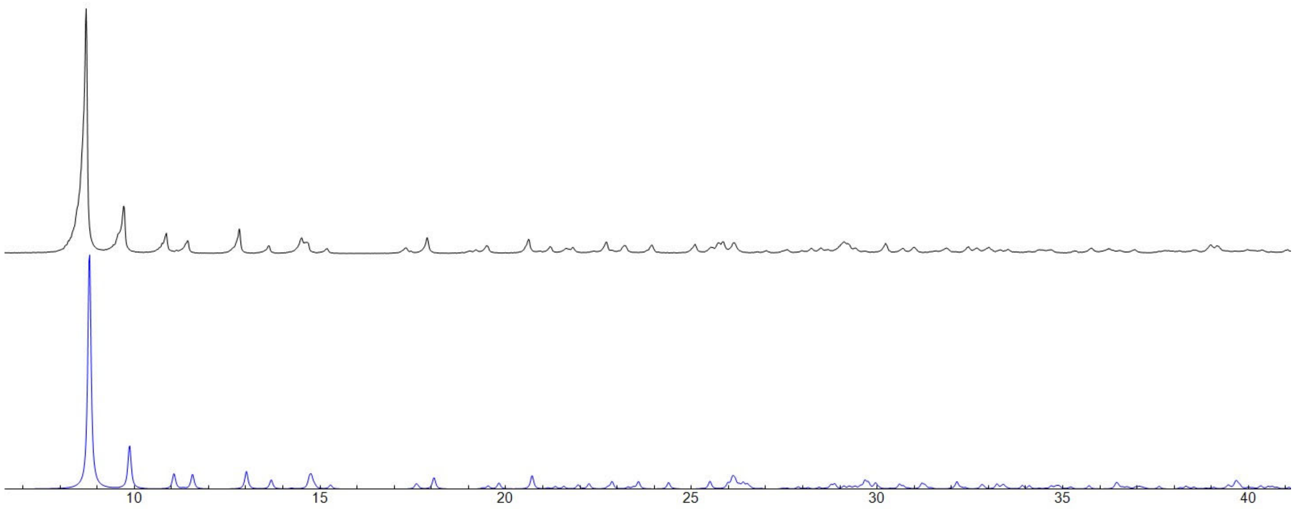

Figure S7. Visual comparison of the experimental powder pattern of [$(CuBr)_4${*cyclo*-(P_3*t*Bu_3)}$_2$] (**2**) (black, top) and the pattern simulated from the single crystal structure model recorded at 130 K (blue, bottom). Abscissa 2Θ in °.

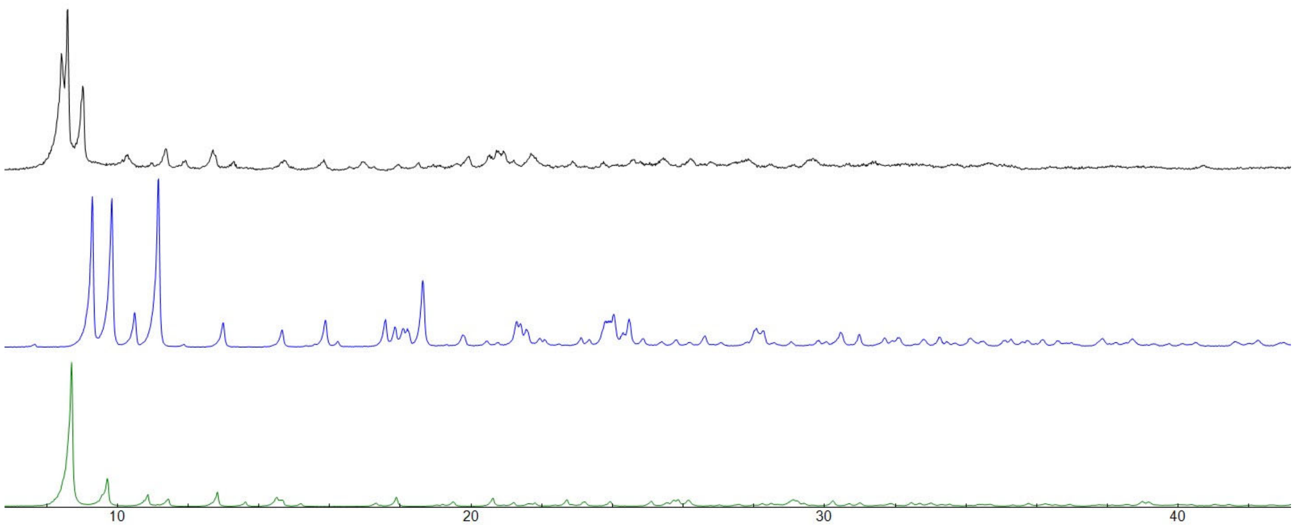

Figure S8. Visual comparison of the experimental powder pattern of the bulk material obtained in the synthesis of complex **3a** (black, top) and patterns for [$(CuBr)_2${*cyclo*-(P_3*t*Bu_3)}$_2$] (**1**) (blue, center) and [$(CuBr)_4${*cyclo*-(P_3*t*Bu_3)}$_2$] (**2**) (green, bottom). Abscissa 2Θ in °.

3. Experimental Section

3.1. General Remarks

All experiments were performed under an atmosphere of dry nitrogen using standard Schlenk techniques. The solvents toluene, dichloromethane, diethyl ether, *n*-pentane, *n*-hexane and acetonitrile were dried and degassed with an MB SPS-800 Solvent Purification System (MBRAUN). Acetonitrile was distilled from calcium hydride. THF was distilled from potassium and benzophenone. All solvents were kept over activated molecular sieves (4 Å). The phosphane *cyclo*-(P_3*t*Bu_3) was synthesized according to literature methods.[53]

Samples for IR spectroscopy were prepared as KBr pellets in a nitrogen-filled glove box and the spectra were recorded on a PerkinElmer (System 2000) FTIR spectrometer in the range of 350-4000 cm^{-1}. Elemental analyses (EA) for C, H, and N were performed on a FlashEA1112 element analyzer. Thermogravimetric (TG) and differential thermal analysis (DTA) curves, coupled with mass spectrometry, were obtained using a NETZSCH STA449F1 thermoanalyzer in a dynamic argon atmosphere (heating rate 10 °C·min^{-1}, flow rate 25 ml·min^{-1}, aluminum oxide crucible, mass about 20 mg, and temperature range from room temperature up to 900 °C).

3.2. Synthesis of Complex 1

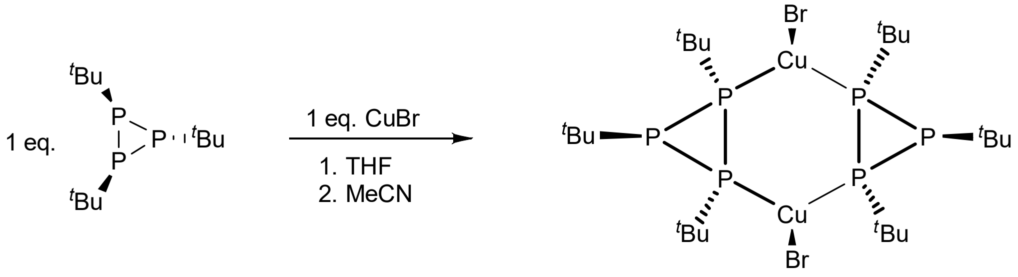

1

At room temperature, 10.3 ml (5.05 mmol, 1.0 eq.) of a 0.49 molar solution of *cyclo*-($P_3t$$Bu_3$) in THF were added to a suspension of CuBr (725 mg, 5.05 mmol, 1.0 eq.) in 100 ml THF. After stirring overnight, the solvent was removed, and the residue was dissolved in 40 ml boiling acetonitrile. The hot solution was filtered with a filter canula, and the filtrate was cooled first to room temperature and after one day to 4 °C. The formed crystals were isolated by filtration and solvent traces were removed in high vacuum. 1.500 g (1.84 mmol, yield 73%) of **1** were obtained as slightly yellow crystals.

EA: Found: C = 35.41%, H = 6.32%, N = 0.0%;
Calculated: C = 35.35%, H = 6.68%, N = 0.0%.
HRMS (ESI, acetonitrile, positive mode):
Found: m/z = 735.041; calculated for $[M–Br]^+$ = 735.041.
IR (ATR): $\tilde{\nu}$ = 2954 (m), 2887 (m), 2856 (m), 1457 (s), 1392 (w), 1363 (s), 1197 (w), 1161 (s), 1012 (m), 939 (w), 804 (m), 597 (w), 536 (w), 442 (w) cm^{-1}.
NMR (25 °C):
^{1}H NMR (400 MHz, $CDCl_3$) δ = 1.52 (bs, 36H), 1.30 (bd, $^3J_{H\text{-}P}$ = 15.2 Hz, 18H) ppm.
^{31}P{^{1}H} NMR (162 MHz, $CDCl_3$) δ = -54.3 to - 66.5 (d), -95.5 to -105.0 (t) ppm.
^{31}P NMR (162 MHz, $CDCl_3$) δ = -52.7 to - 68.0 (d), -95.3 to -105.5 (t) ppm.
^{13}C NMR (101 MHz, $CDCl_3$) δ = 32.8 (bs, C−C−P), 31.0 (pseudo-d, $^2J_{C\text{-}P}$ = 16.4 Hz, C−P) ppm.

NMR (–60 °C):
^{1}H NMR (400 MHz, $CDCl_3$) δ = 1.59 (bs), 1.51 (bs), 1.32 (bd, $^3J_{H\text{-}P}$ = 15.2 Hz) ppm.
^{31}P{^{1}H} NMR (162 MHz, $CDCl_3$) AA'BB'B''B''' spin system (see Figure 2) with C_{2v} symmetry; $\delta(P_A)$ = -68.2459(2) ppm, $\delta(P_B)$ = -106.864(1) ppm, $H(P_A)$ = 21.5(2) Hz, $H(P_B)$ = 20.95(9) Hz, $^1J_{AB}$ = –211.4(1) Hz, $^1J_{BB'}$ = –182(3) Hz, $^2J_{BB''}$ = +139.8(4) Hz, $^3J_{AB'}$ = –1.4(1)Hz, $^3J_{BB'''}$ = –14.6(5)Hz, $^4J_{AA'}$ = +6.5(6) Hz (R = 0.87%).

3.1. Synthesis of Complex 2

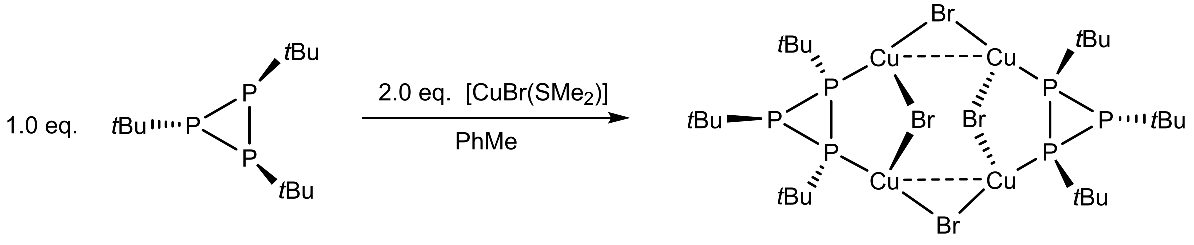

2

At room temperature, 3.5 ml (2.03 mmol, 1.0 eq.) of a 0.58 molar solution of *cyclo*-(P_3tBu_3) in toluene were added to a suspension of [CuBr(SMe_2)] (833 mg, 4.05 mmol, 2.0 eq.) in 40 ml toluene. After stirring overnight, the solvent was removed at reduced pressure and the residue was dissolved in 50 ml boiling toluene. The hot solution was filtered with a filter canula, and the filtrate was cooled to rt and after one day to 4 °C. The formed crystals were isolated by filtration and solvent traces were removed in high vacuum at 50 °C. 940 mg (0.85 mmol, yield 84%) of **2** were obtained as slightly yellow crystals.

EA: Found: C = 26.73%, H = 4.97%, N = 0%;
Calculated: C = 26.15%, H = 4.94%, N = 0%.
HRMS (ESI, MeCN, positive mode):
Found: m/z = 1022.733; calculated for $[M–Br]^+$ = 1022.733.
^{1}H NMR (400 MHz, $CDCl_3$) δ = 1.46 (t, $^3J_{H\text{-}P}$ = 9.1 Hz, 36H), 1.31 (d, $^3J_{H\text{-}P}$ = 15.4 Hz, 18H) ppm.
^{31}P{^{1}H} NMR (162 MHz, $CDCl_3$) δ = -55.7 (d, $^1J_{P\text{-}P}$ = 227.4 Hz), -90.7 (t, $^1J_{P\text{-}P}$ = 227.4 Hz) ppm.
^{31}P NMR (162 MHz, $CDCl_3$) δ = -52.6 to -58.1 (m), -88.7 to -92.8 (m) ppm.
^{13}C{^{1}H} NMR (101 MHz, $CDCl_3$) δ = 37.1 to 36.6 (m), 32.0 to 31.6 (m), 31.4 to 31.1 (m), 29.9 (d, $^1J_{C\text{-}P}$ = 44.0 Hz) ppm.

3.2. Synthesis of Complex 3a

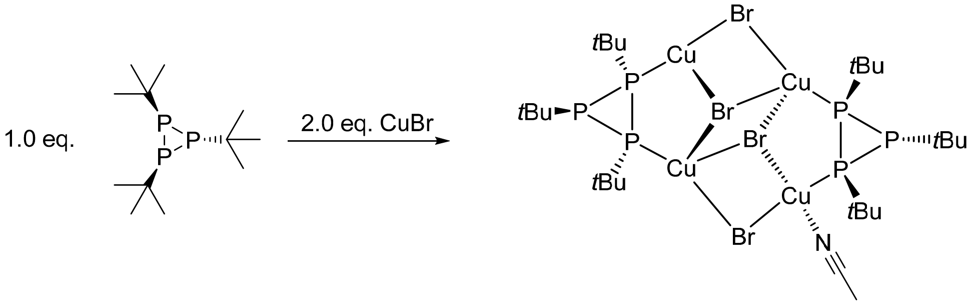

3a

At room temperature, 8.0 ml (3.84 mmol, 1.0 eq.) of a 0.49 molar solution of *cyclo*-(P_3tBu_3) in THF were added to a suspension of CuBr (1100 mg, 7.67 mmol, 2.0 eq.) in 70 ml THF. After stirring overnight, the solvent was removed and the residue was dissolved in 80 ml boiling acetonitrile. The hot solution was filtered with a filter canula and the filtrate was cooled to rt and after one day to 4 °C. The formed crystals

were isolated by filtration and solvent traces were removed in high vacuum. 1.740 g (1.52 mmol, yield 79%) of **3a** were obtained as slightly yellow crystals.

The acetonitrile content was determined via elemental analysis and varied. The freshly synthesized compound contained on average about one acetonitrile molecule per complex. A sample of this substance was recrystallized from acetonitrile and subsequently contained about two acetonitrile ligands per complex. An investigation by X-ray structure analysis resulted in the same cell parameters in all cases.

EA after synthesis: Found: C = 26.92%, H = 4.94%, N = 0.98%;
Calculated: C = 27.31%, H = 5.03%, N = 1.23%.
EA after additional recrystallisation: Found: C = 28.19%, H = 4.96%, N = 2.13%;
Calculated: C = 28.39%, H = 5.11%, N = 2.37%.
HRMS (ESI, acetonitrile, positive mode):
Found: m/z = 1022.733; calculated for $[M–Br–2\ MeCN]^+$ = 1022.733.
IR (KBr): $\tilde{\nu}$ = 2949 (s), 2889 (m), 2855 (m), 1454 (s), 1395 (w), 1364 (w), 1195 (w), 1161 (s), 1014 (m), 932 (w), 803 (m), 579 (w), 538 (w), 444 (w) cm^{-1}.
^{1}H NMR (400 MHz, $CDCl_3$) δ = 2.01 (s, MeCN), 1.47 (t, $^3J_{H\text{-}P}$ = 9.1 Hz, 36H), 1.31 (d, $^3J_{H\text{-}P}$ = 15.4 Hz, 18H) ppm.
^{31}P{^{1}H} NMR (162 MHz, $CDCl_3$) δ = -55.5 (d, $^1J_{P\text{-}P}$ = approx. 225 Hz), -90.7 (t, $^1J_{P\text{-}P}$ = 227.2 Hz) ppm.
^{31}P{^{1}H} NMR (162 MHz, C_6D_6) δ = -55.7 (d, $^1J_{P\text{-}P}$ = approx. 220 Hz), -90.0 (t, $^1J_{P\text{-}P}$ = 224.9 Hz) ppm.
^{31}P NMR (162 MHz, C_6D_6) δ = -53.4 to -57.5 (m), -89.0 to -92.9 (m) ppm.
^{13}C{^{1}H} NMR (101 MHz, $CDCl_3$) δ = 116.3 (s, CH_3**C**N), 37.1 to 36.6 (m), 31.9 to 31.6 (m), 31.4 to 31.1 (m), 29.9 (d, $^1J_{C\text{-}P}$ = 43.7 Hz), 1.9 (s, **C**H_3CN) ppm.

Table S3. NMR titration experiment. Complex **3a** showed a singlet in the ^{1}H NMR spectrum ($CDCl_3$) at 2.01 ppm, which can be assigned to the acetonitrile acting as a co-ligand in the solid state. Aliquots of acetonitrile were added to a solution of **3a** to verify whether the acetonitrile molecule is coordinated or dissociated from the copper atom in solution.

V solution of **3a** in $CDCl_3$ c = 12 mmol/l	n **3a**	eq. **3a**	V solution of MeCN in $CDCl_3$ c = 76 mmol/l	n MeCN	eq. MeCN	Ratio of the integrals C**H**$_3$CN / P_3***t*****Bu**$_3$	
						measured	calculated
1 ml	12 µmol	1		0	0	1.9 / 54	-
1 ml	12 µmol	1	160 µl	12 µmol	1	3.0 / 54	4.9 / 54
1 ml	12 µmol	1	470 µl	36 µmol	3	5.0 / 54	10.9 / 54

3.3. Synthesis of Complex 3b

At room temperature a solution of *cyclo*-($P_3t$$Bu_3$) (0.27 g, 1.02 mmol) in THF (2 ml) was added dropwise to a solution of CuBr (0.144 g, 1.0 mmol) in THF (25 ml). The yellow solution turned greenish during the addition and a white precipitate formed. The reaction mixture was stirred at room temperature overnight and the solvent was removed *in vacuo* to give a white precipitate. Half of this quantity was dissolved in hot THF (10 mL) and cooled to –15 °C. After 4 hours, colorless crystals of **3b** formed (689.0 mg, 54% with respect to half the quantity), which were suitable for single-crystal X-ray diffraction.

EA: Found: C = 30.19%, H = 5.14%, N = 0%;

Calculated: C = 30.83%, H = 5.66%, N = 0%.

IR (KBr): ṽ = 3444 (br), 2953 (s, ν_{as}(C–H)), 2889 (s, ν_s(C–H)), 2777 (s), 1455 (vs, $\delta_{as}(CH_3)$), 1393 (w, $\delta_s(CH_3)$), 1364 (vs, $\delta_s(CH_3)$), 1151 (s, $\rho(CH_3$ + ν_s(C–C)), 1017 (s, $\rho(CH_3)$), 936 (w, $\rho(CH_3)$ + ν_s(C–C)), 803 (m, ν_s(C–C)), 536 (w), 444 (w) cm^{-1}.

^{1}H NMR ($CDCl_3$): δ = 1.24 (m, 9H, (**H_3**C)$_3$C-P_B) and 1.40 (m, 18H, (**H_3**C)$_3$C-P_A) ppm.

^{31}P{^{1}H} NMR ($CDCl_3$): −55.9 (pseudo-d), −90.8 (pseudo-t) ppm.

3.1. Synthesis of Complex 4

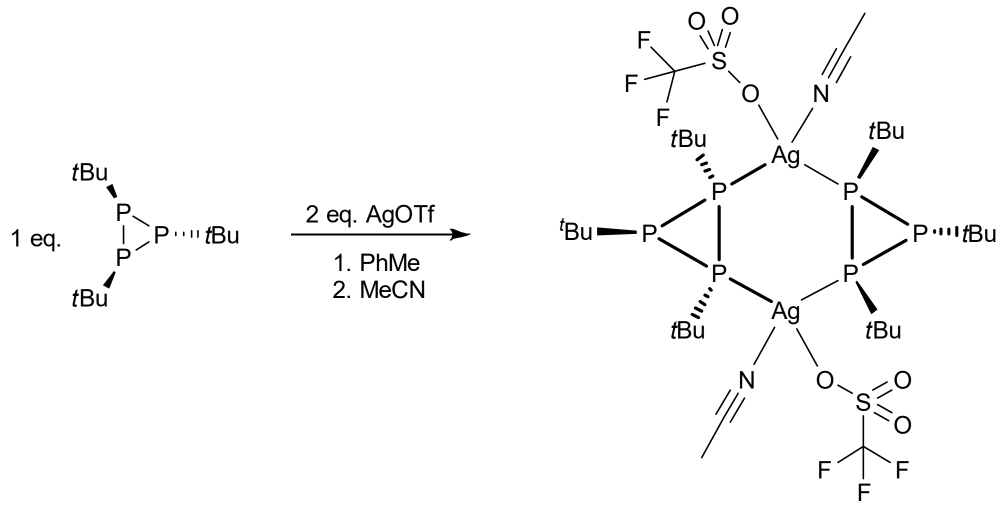

4

At 0 °C, 2.41 ml (1.4 mmol, 1.0 eq.) of a 0.58 molar solution of *cyclo*-($P_3t$$Bu_3$) in toluene were added to a solution of silver(I) triflate (718 mg, 2.8 mmol, 2.0 eq.) in 30 ml toluene. The cooling bath was removed, and the mixture was stirred overnight. The solvent was removed, and the residue was dissolved in 10 ml boiling acetonitrile. The hot solution was filtered with a filter canula, and the filtrate was concentrated to half of the solvent volume at reduced pressure. After storing at –30 °C, the formed crystals were isolated by filtration and solvent traces were removed in high vacuum. 362 mg (0.32 mmol, yield 46%) of **4** were obtained as colorless crystals.

EA: Found: C = 31.10%, H = 5.32%, N = 2.07%;
Calculated: C = 32.04%, H = 5.38%, N = 2.49%.

HRMS (ESI, acetonitrile, positive mode):
Found: m/z = 893.028; calculated for [M–OTf–2 MeCN]$^+$ = 893.027.

IR (ATR): $\tilde{\nu}$ = 2929 (m), 2892 (m), 2862 (m), 2301 (w), 2264 (w), 1458 (w), 1396 (w), 1366 (m), 1287 (s), 1243 (s), 1222 (s), 1158 (s), 1047 (w), 1027 (s), 923 (w), 802 (w), 756 (w), 658 (w), 636 (s), 573 (w), 516 (m) cm^{-1}.

^{1}H NMR (400 MHz, acetonitrile-*d*$_3$) δ = 1.96 to 1.92 (m, 6H), 1.45 to 1.38 (m, 36H), 1.34 (d, J = 15.7 Hz, 18H) ppm.

^{31}P{^{1}H} NMR (162 MHz, acetonitrile-*d*$_3$) δ = -61.9 (d, J = 218.9 Hz), -123.7 (t, J = 218.9 Hz) ppm.

^{31}P NMR (162 MHz, acetonitrile-*d*$_3$) δ = -62.0 (d, J = 218.9 Hz), -123.7 (t, J = 218.9 Hz) ppm.

^{13}C{^{1}H} NMR (101 MHz, acetonitrile-*d*$_3$) δ = 120.3 (q, $^1J_{C\text{-}F}$ = 320.8 Hz, **C**F_3), 36.5 to 35.9 (m, **C quat.**), 30.3 (s, **C**H_3), 28.5 to 28.1 (m, **C**H_3), 28.4 to 27.8 (m, **C quat.**), 0.3 to -1.1 (m, **C**H_3CN) ppm.

3.2. Synthesis of Complex 5

A solution of *cyclo*-(P_3tBu_3) (0.135 g, 0.51 mmol) in toluene (10 ml) was added dropwise to a solution of $Ag(CF_3SO_3)$ (0.262 g, 1.0 mmol) in toluene (30 ml) at 0 °C. The clear colorless solution turned turbid during the addition. The reaction mixture was left to warm to room temperature and stirred overnight. The solvent was removed *in vacuo*. The residue was dissolved in hot MeCN (10 mL) and then cooled to −15 °C. Colorless needle-shaped crystals of **5** suitable for single-crystal X-ray diffraction were obtained after 6 days (483.0 mg, 53%).

EA: Found: C = 27.34%, H = 4.53%, N = 4.28%;
Calculated: C = 26.65%, H = 4.03%, N = 4.66%.

IR (KBr) $\tilde{\nu}$ = 3421 (w, br), 2952 (s, ν_{as}(C–H)), 2890 (s, ν_s(C–H)), 2293 (sh), 2264 (w), 1459 (s, $\delta_{as}(CH_3)$), 1394 (w, $\delta_s(CH_3)$), 1366 (vs, $\delta_s(CH_3)$), 1261 (vs), 1162 (s, $\rho(CH_3 + \nu_s(C–C))$, 1040 (s, $\rho(CH_3)$), 935 (w, $\rho(CH_3) + \nu_s$(C–C)), 803 (m, ν_s(C–C)), 641 (vs), 577 (w), 519 (s) cm^{-1}.

^{1}H NMR (CD_3CN): δ = 1.26 (m, 18H, (**H**$_3$C)$_3$C-P$_B$),1.3 (br, s, 36H, (**H**$_3$C)$_3$C-P$_A$), 2.11 (s, 18H, **H**$_3$C-CN) ppm.

^{31}P{^{1}H} NMR (CD_3CN): −61.3 (pseudo-d), −123.0 (pseudo-t) ppm.

4. Quantum Chemical Calculations

All calculations were carried out with DFT using the program ORCA[54] (version 5.0.1). For all calculations, the convergence criterion was set to tight SCF convergence and the atom-pairwise dispersion correction based on tight binding partial charges (D4)[55,56] was used. Furthermore, the RIJDX approximation was used to speed up the calculations. In all cases, the free Gibbs energy ΔG was calculated using a numerical frequency analysis. In each case, only positive Eigenvalues in the Hesse matrix confirmed an energetic minimum.

5. NMR Measurements and Spectra of the Compounds

The NMR spectra were recorded at 25 °C with a Bruker AVANCE DRX 400 spectrometer (^{1}H NMR: 400.13 MHz, ^{13}C NMR: 101 MHz, ^{31}P NMR: 161.97 MHz). TMS was used as internal standard for ^{1}H NMR spectra. ^{13}C and ^{31}P NMR spectra were referenced to the Ξ scale[57]. Simulation of the NMR spectra was performed using the DAISY module implemented in the program TopSpin version 4.0 (BRUKER, BioSpin GmbH, Rheinstetten).[58] For this, the $^1J_{P-P}$ coupling constants were set negative.[59] For low-temperature NMR experiments, a capillary with methanol-d_4 was added to the NMR sample, which served as an internal thermometer. The temperature was measured indirectly by determining the change in chemical shift of the two signals of the methanol-d_4.[60]

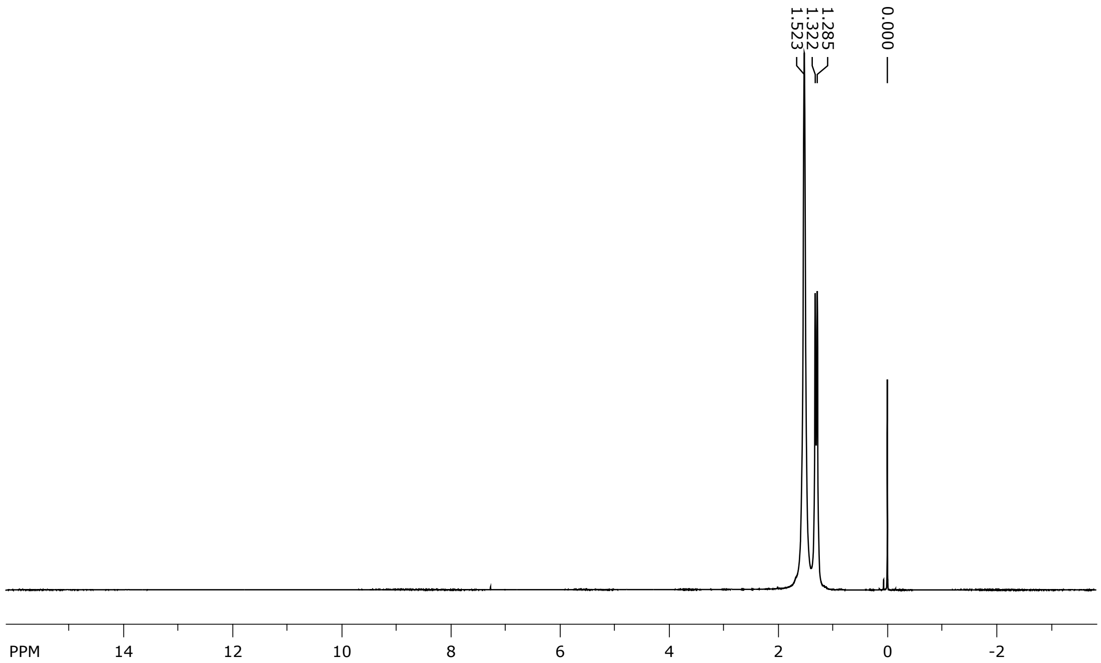

Figure S9. ^{1}H NMR spectrum of complex **1** at *room temperature*.

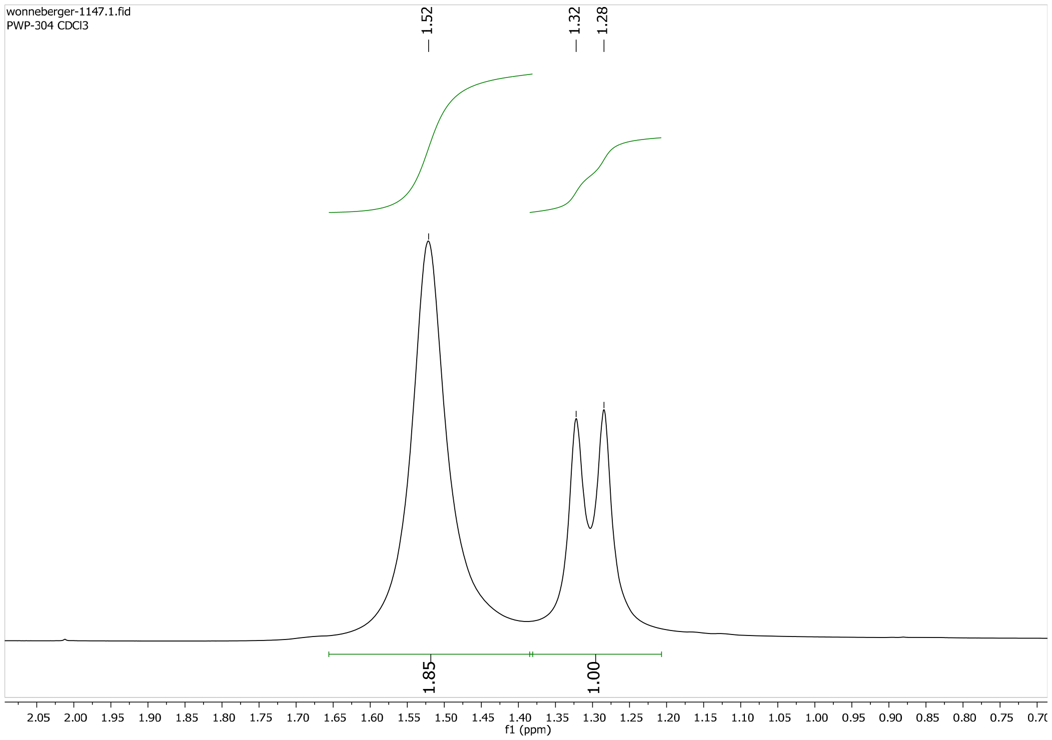

Figure S10. ^{1}H NMR spectrum of complex **1** at *room temperature*.

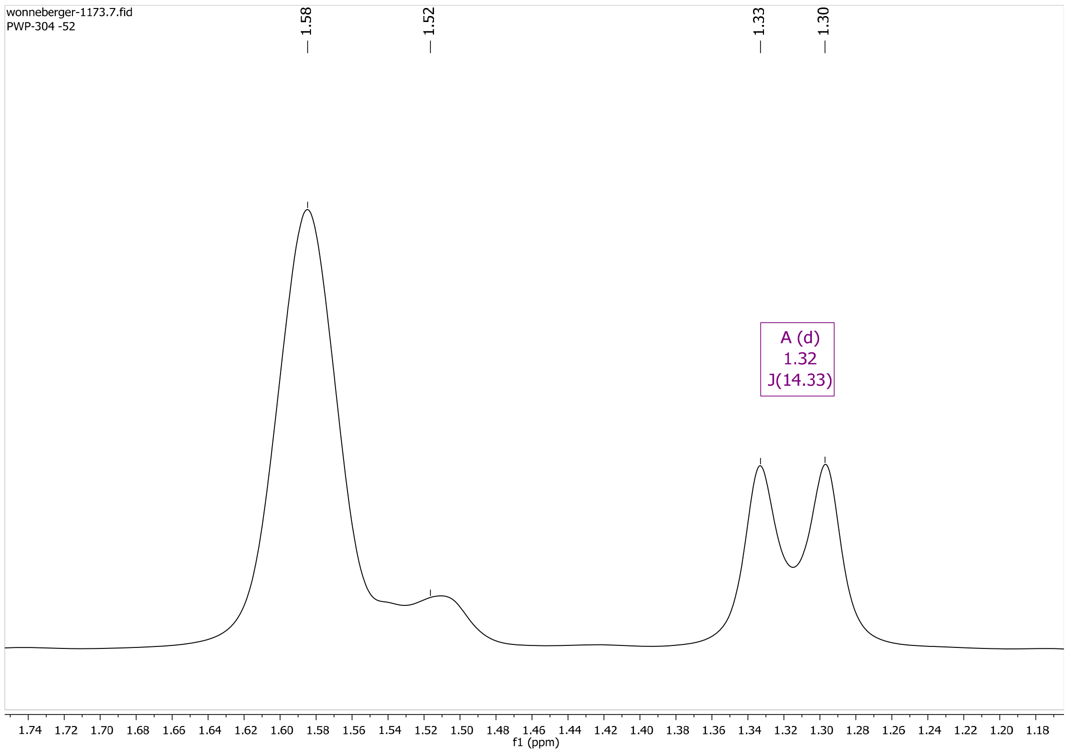

Figure S11. ^{1}H NMR spectrum of complex **1** at −60 °C.

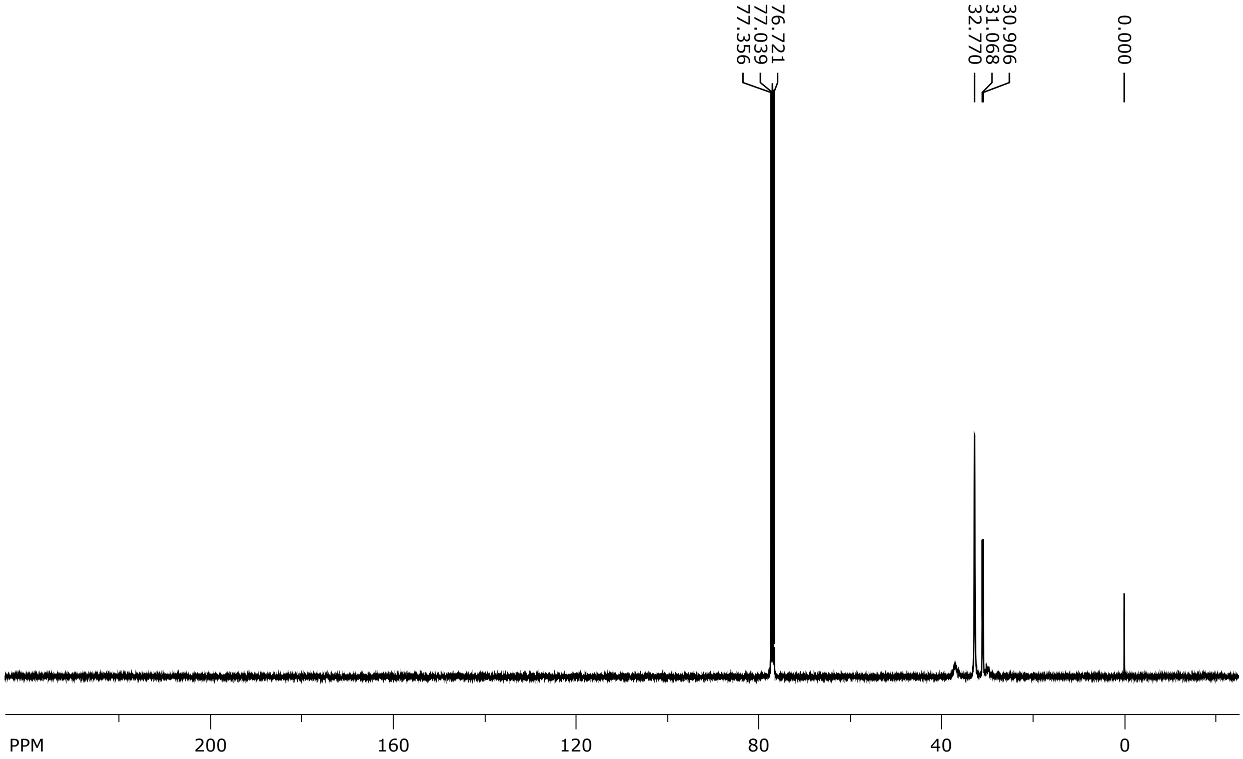

Figure S12. $^{13}C\{^{1}H\}$ NMR spectrum of complex **1** at *room temperature*.

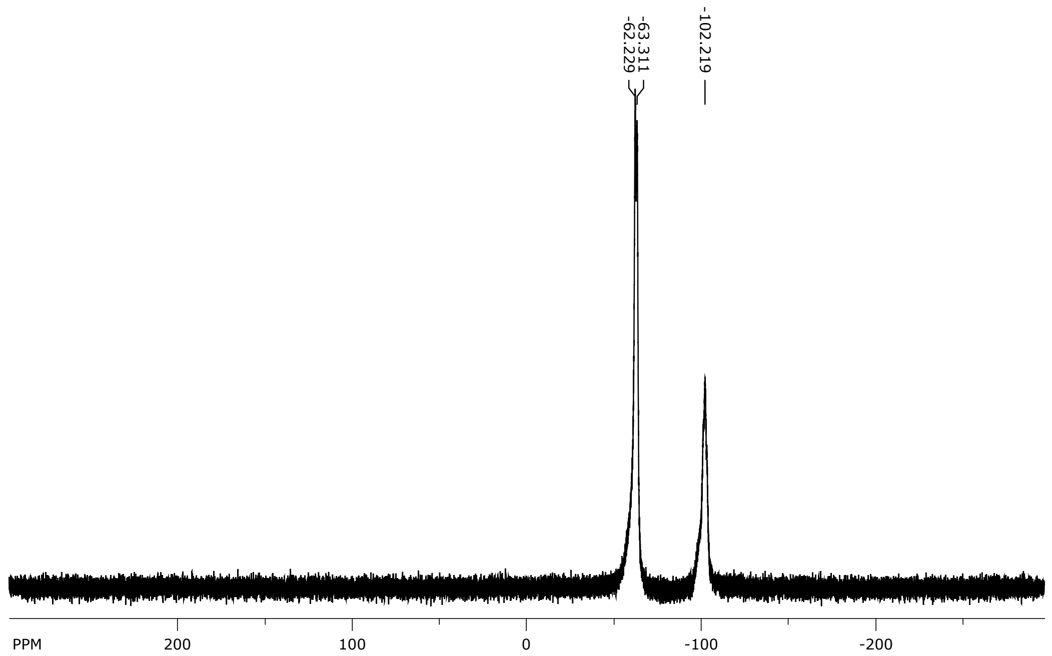

Figure S13. $^{31}P\{^{1}H\}$ NMR spectrum of complex **1** at *room temperature*.

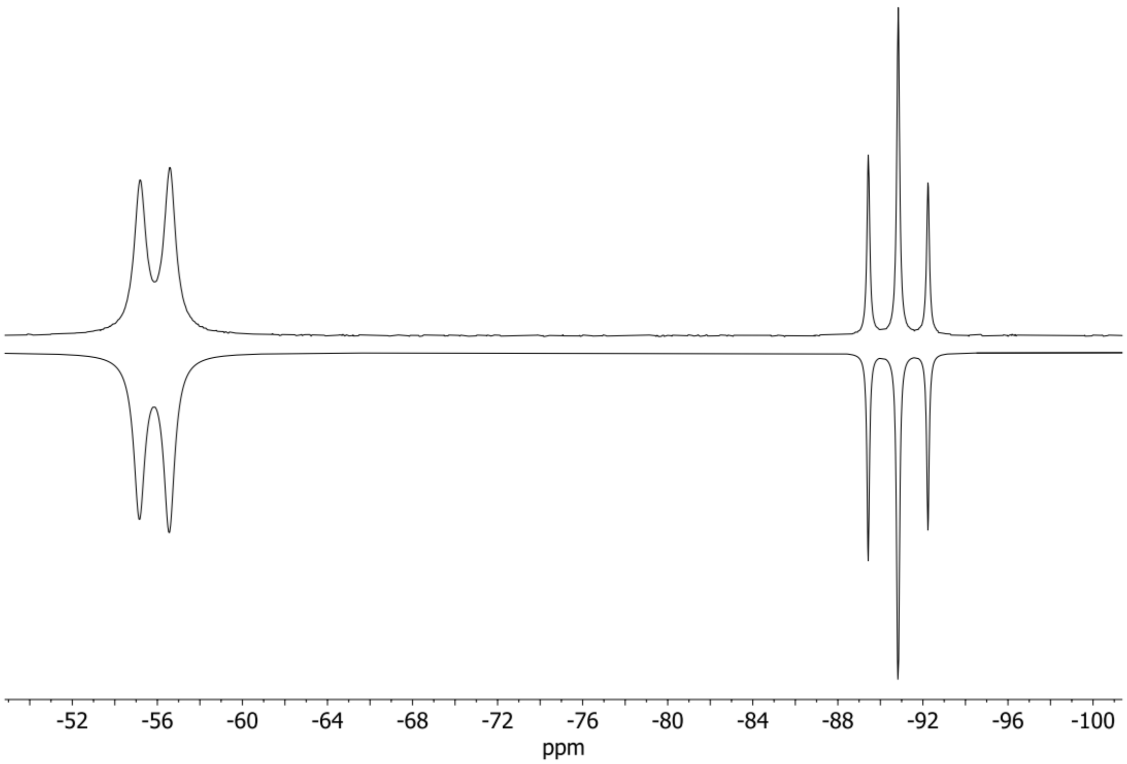

Figure S14. Experimental (top) and simulated (bottom) $^{31}P\{^{1}H\}$ NMR spectrum of complex **1** at *room temperature*.

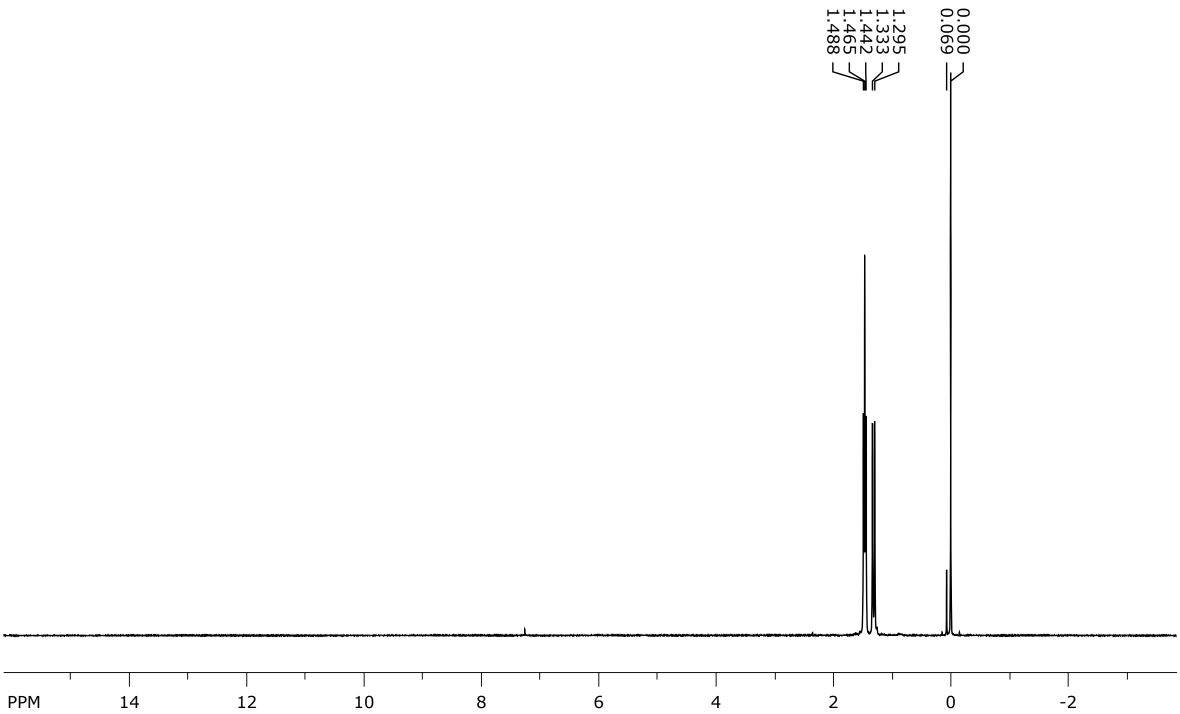

Figure S15. ^{1}H NMR spectrum of complex **2** at *room temperature*.

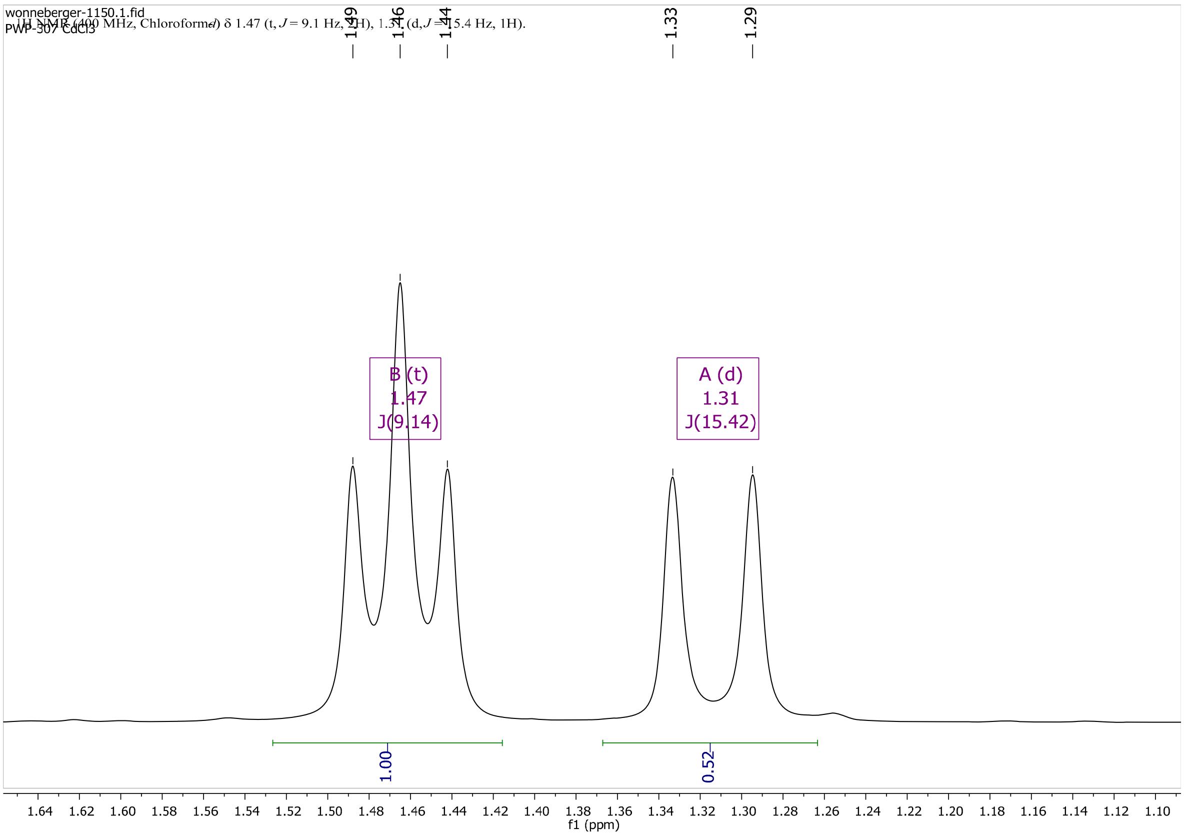

Figure S16. ^{1}H NMR spectrum of complex **2** at *room temperature*.

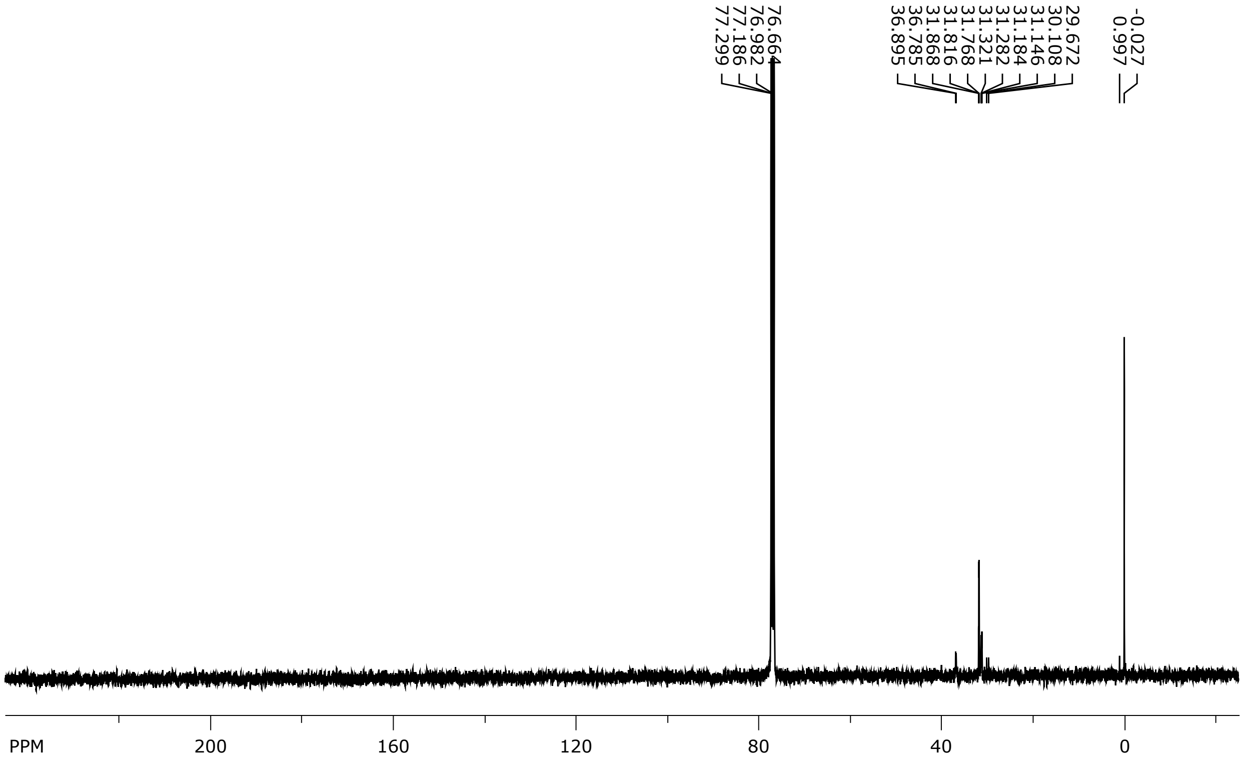

Figure S17. $^{13}C\{^{1}H\}$ NMR spectrum of complex **2** at *room temperature*.

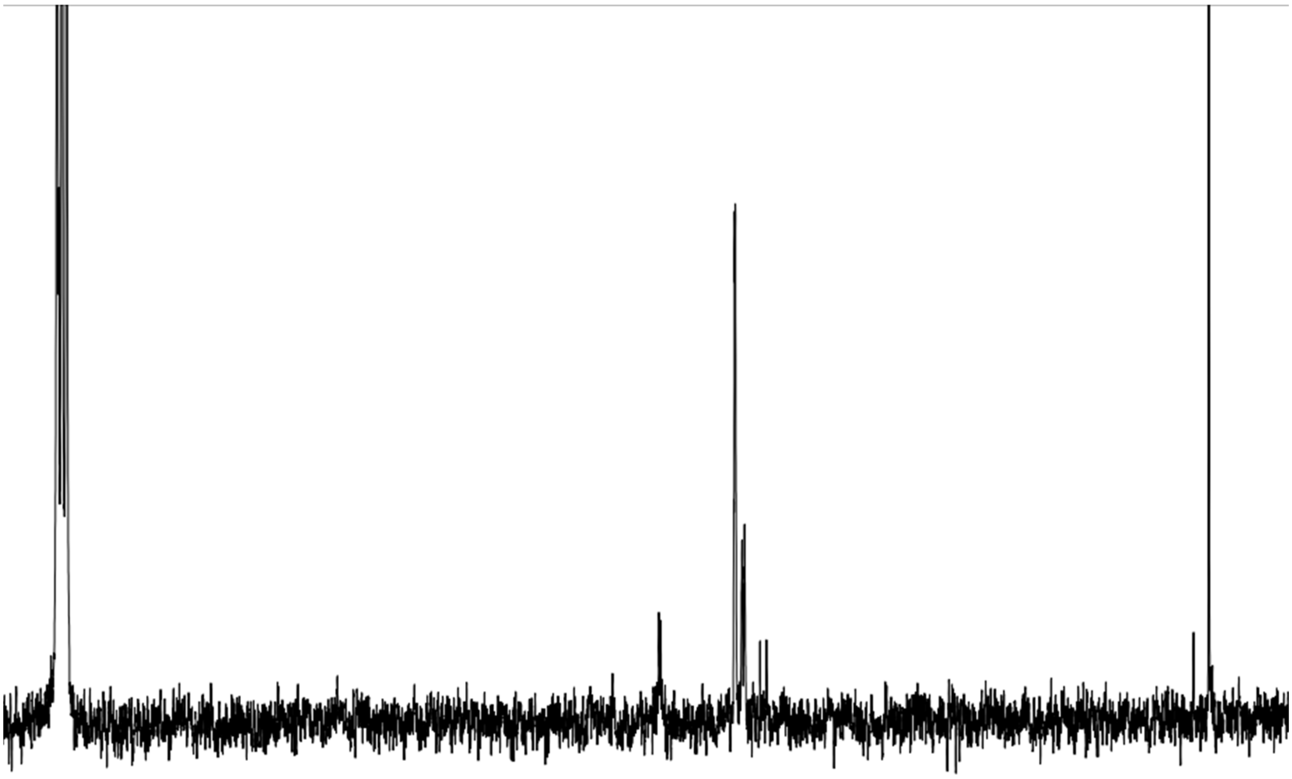

Figure S18. $^{13}C\{^{1}H\}$ NMR spectrum of complex **2** at *room temperature*.

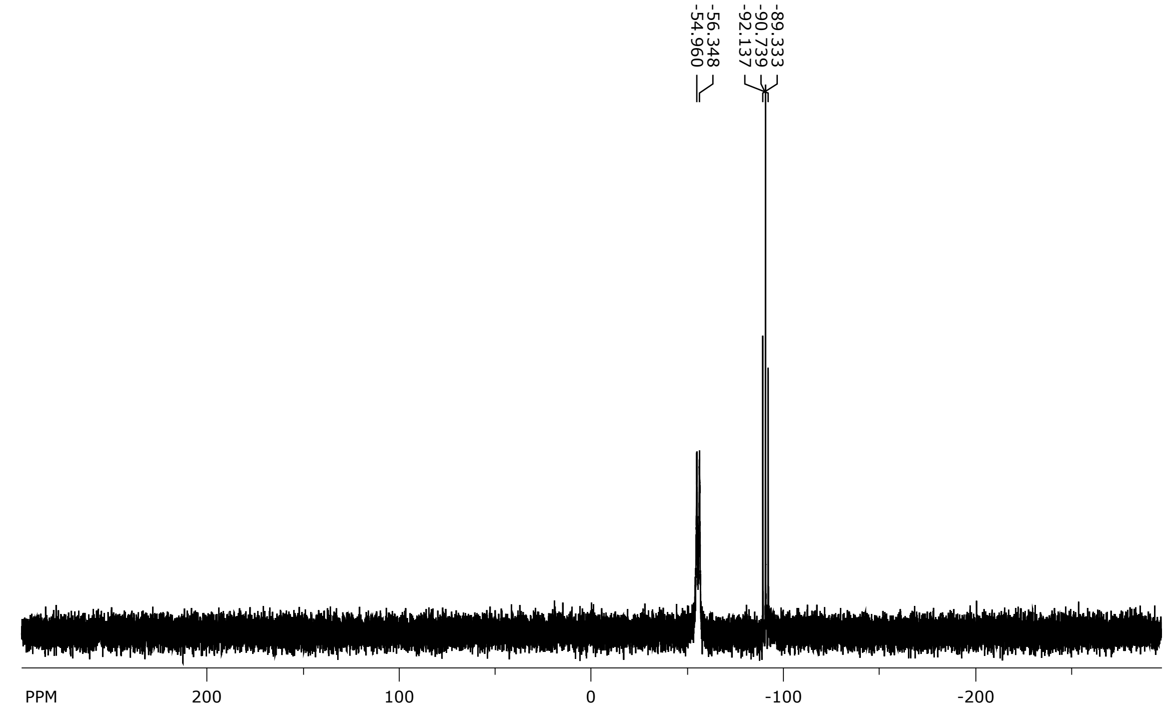

Figure S19. $^{31}P\{^{1}H\}$ NMR spectrum of complex **2** at *room temperature*.

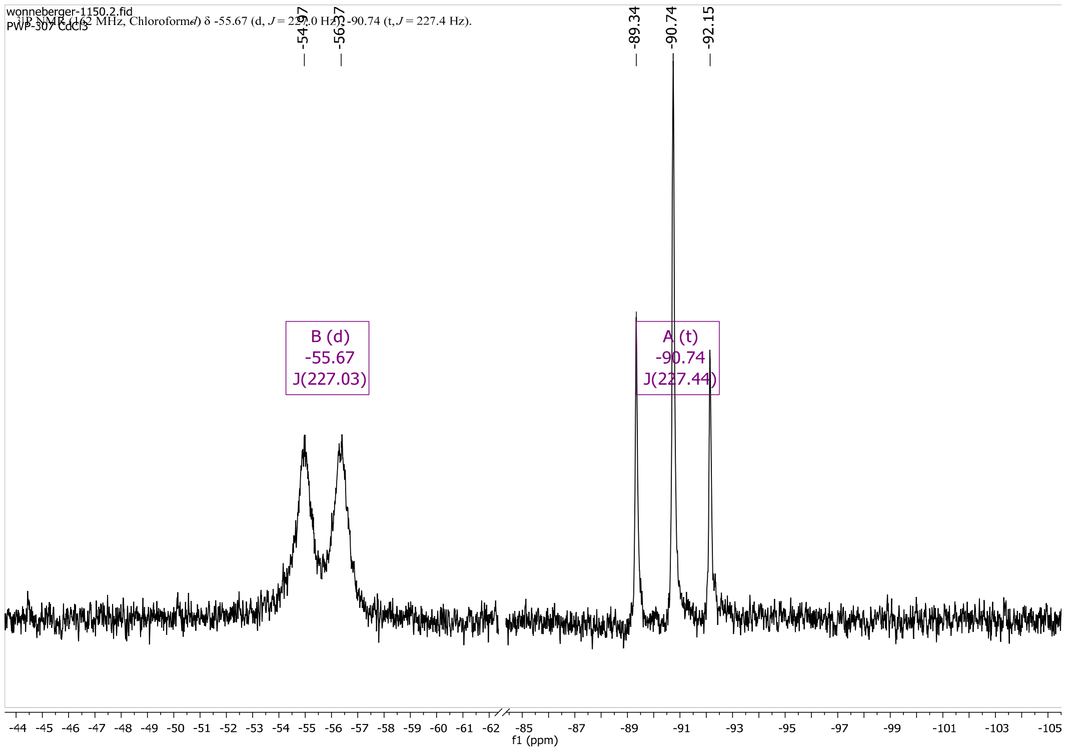

Figure S20. $^{31}P\{^{1}H\}$ NMR spectrum of complex **2** at *room temperature*.

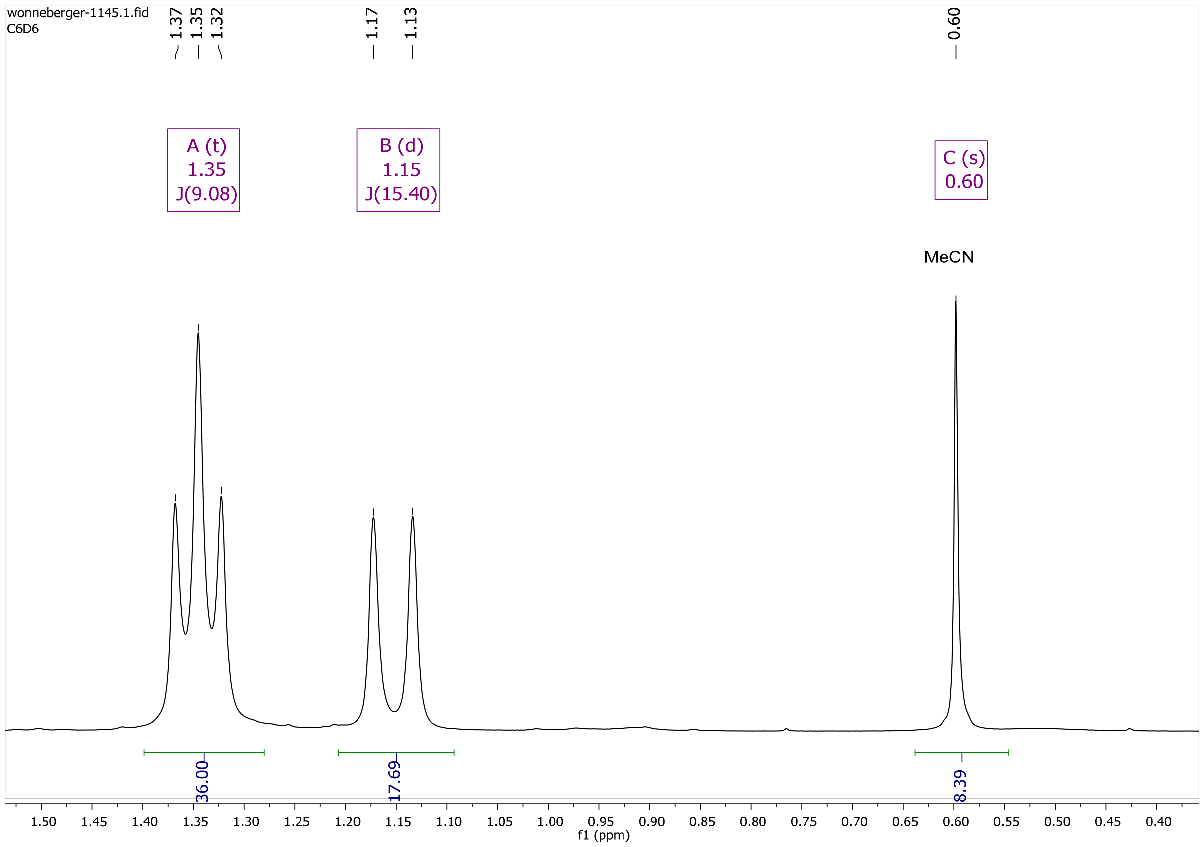

Figure S21. ^{1}H NMR spectrum of complex **3a** at *room temperature*.

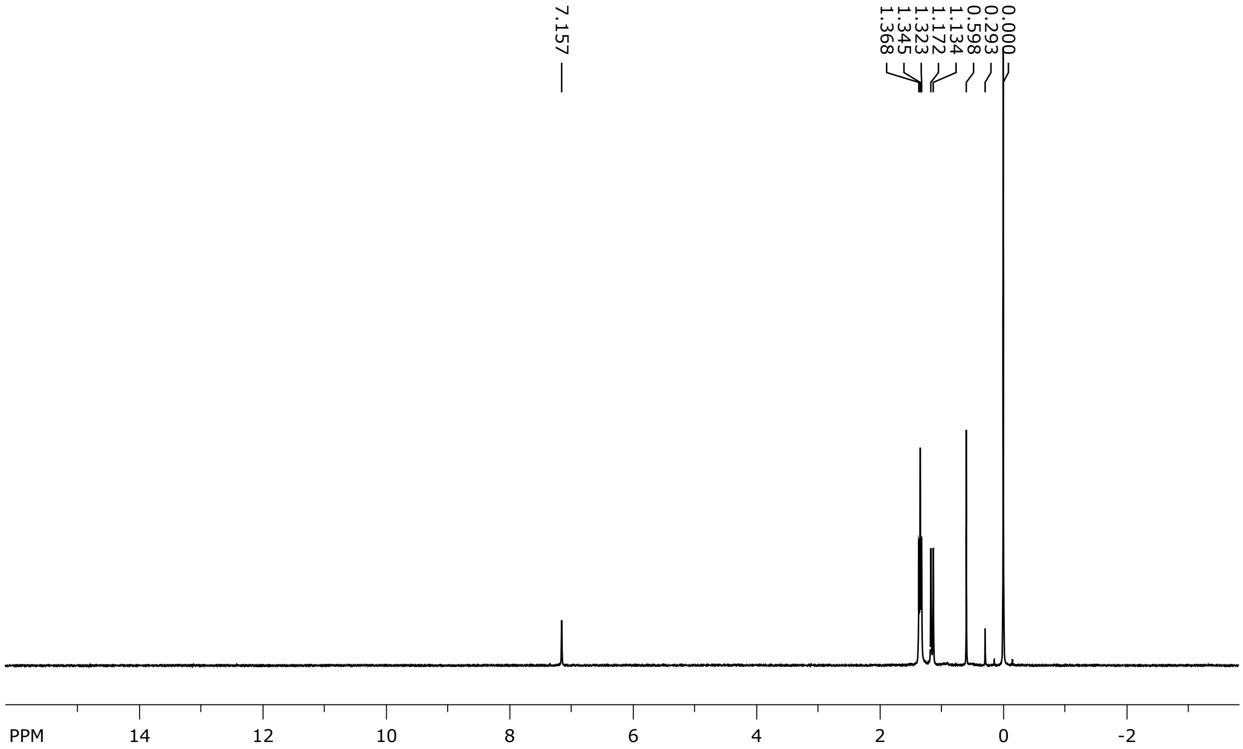

Figure S22. ^{1}H NMR spectrum of complex **3a** at *room temperature*.

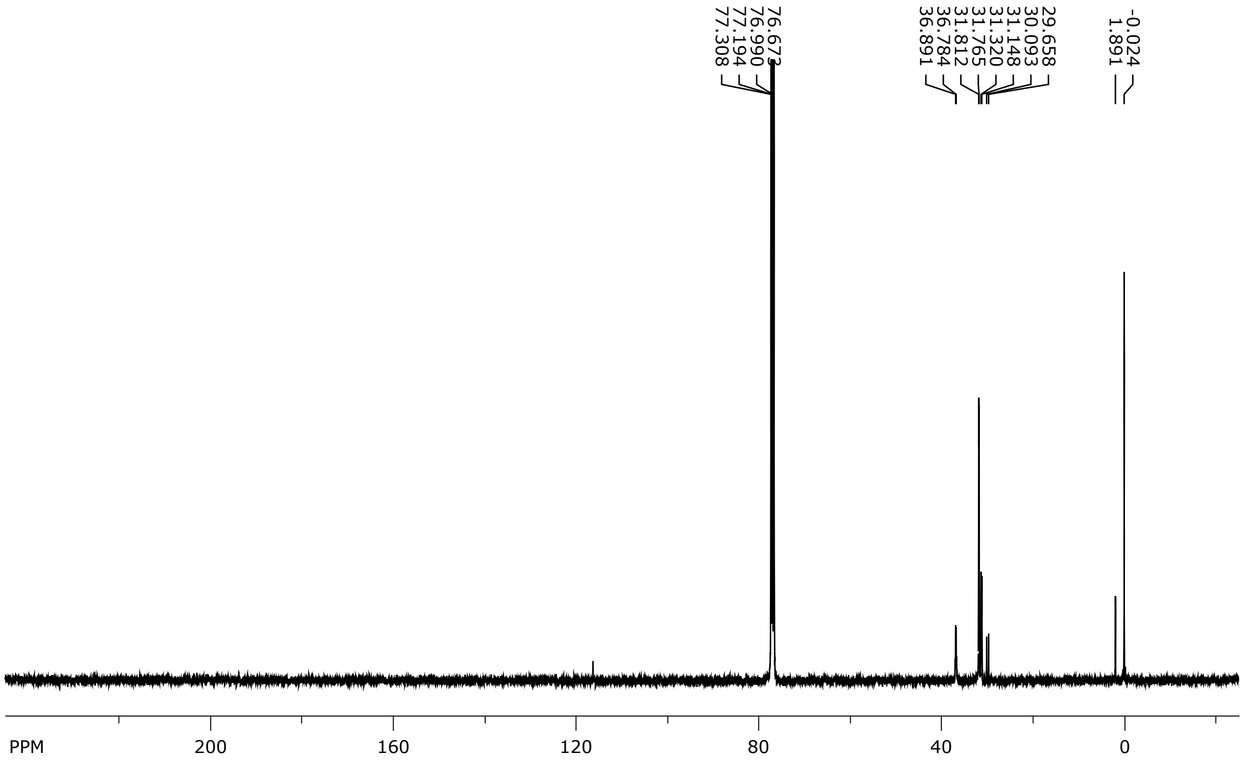

Figure S23. $^{13}C\{^1H\}$ NMR spectrum of complex **3a** at *room temperature*.

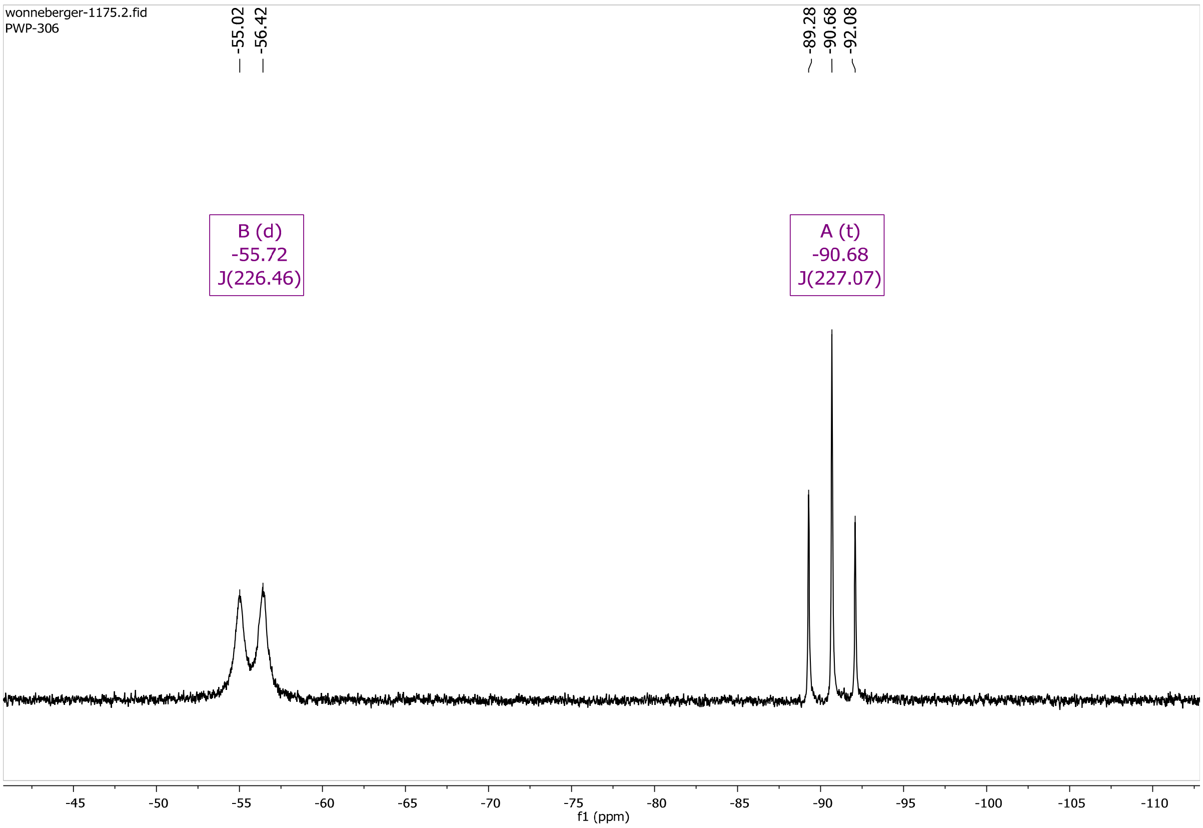

Figure S24. $^{31}P\{^1H\}$ NMR spectrum of complex **3a** at *room temperature*.

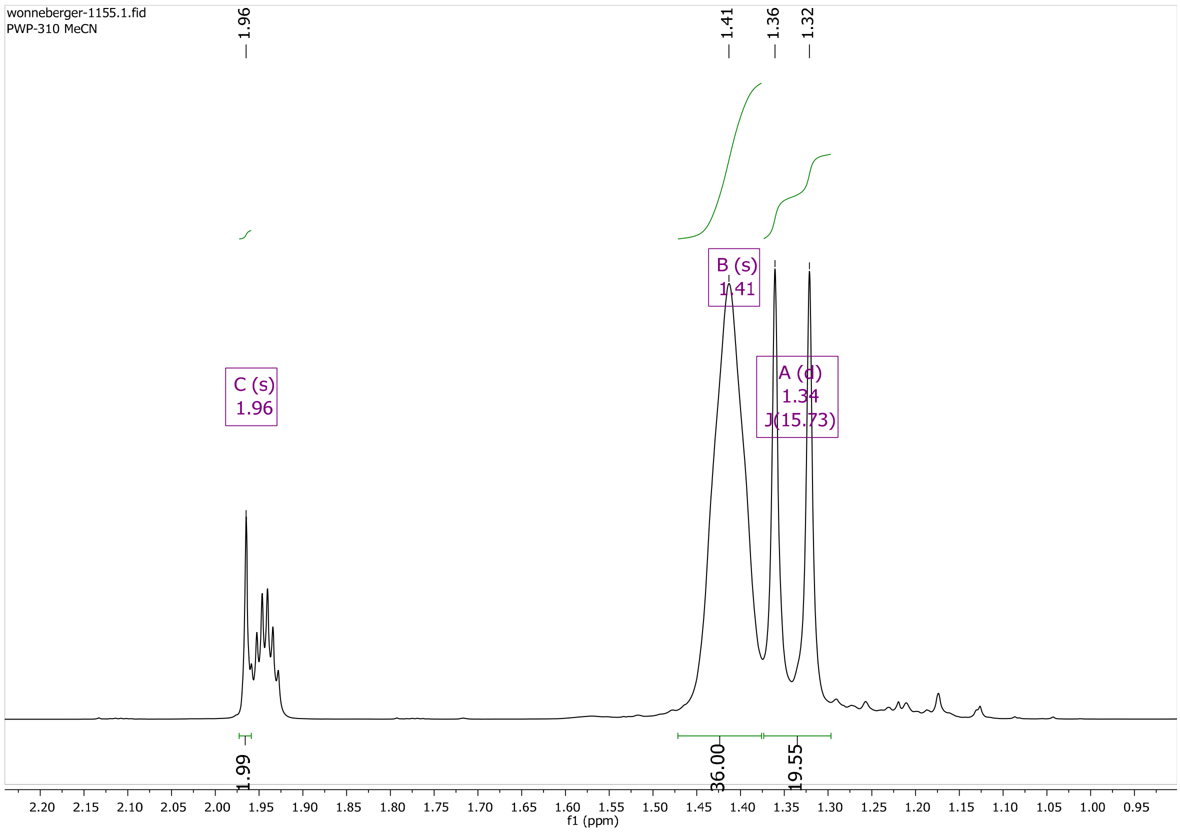

Figure S25. ^{1}H NMR spectrum of complex **4** at *room temperature*.

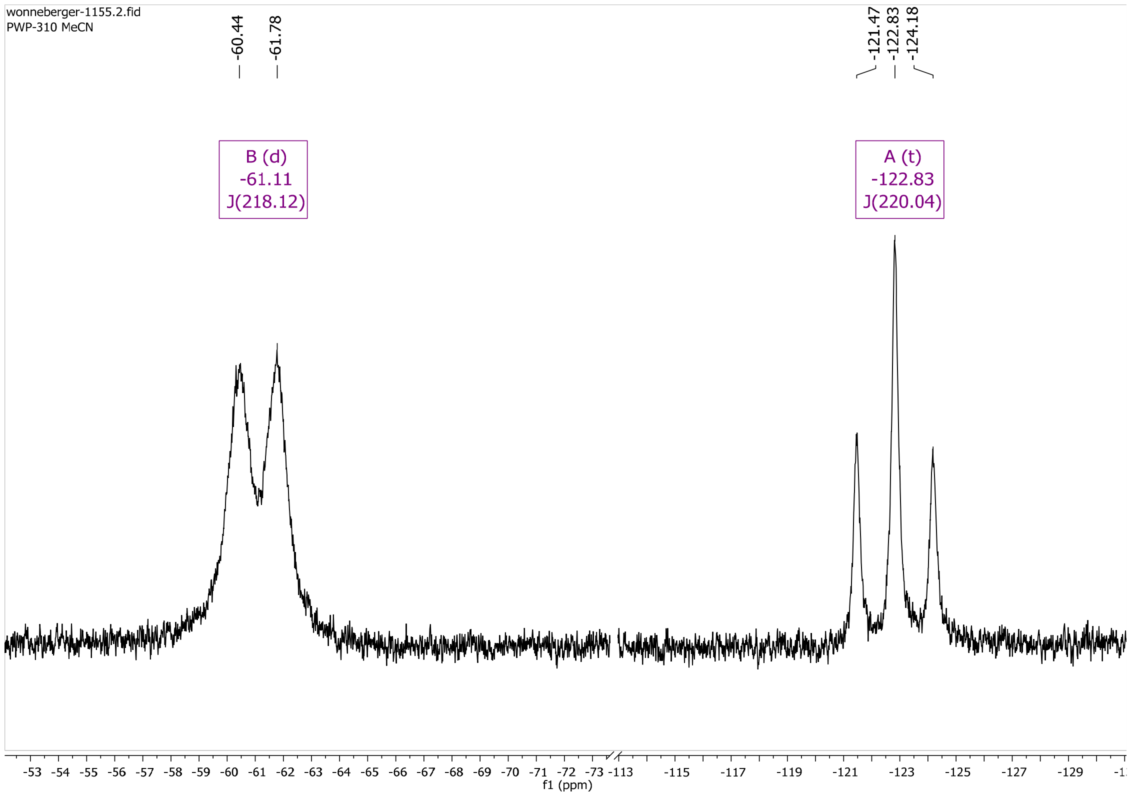

Figure S26. $^{31}P\{^{1}H\}$ NMR spectrum of complex **4** at *room temperature*.

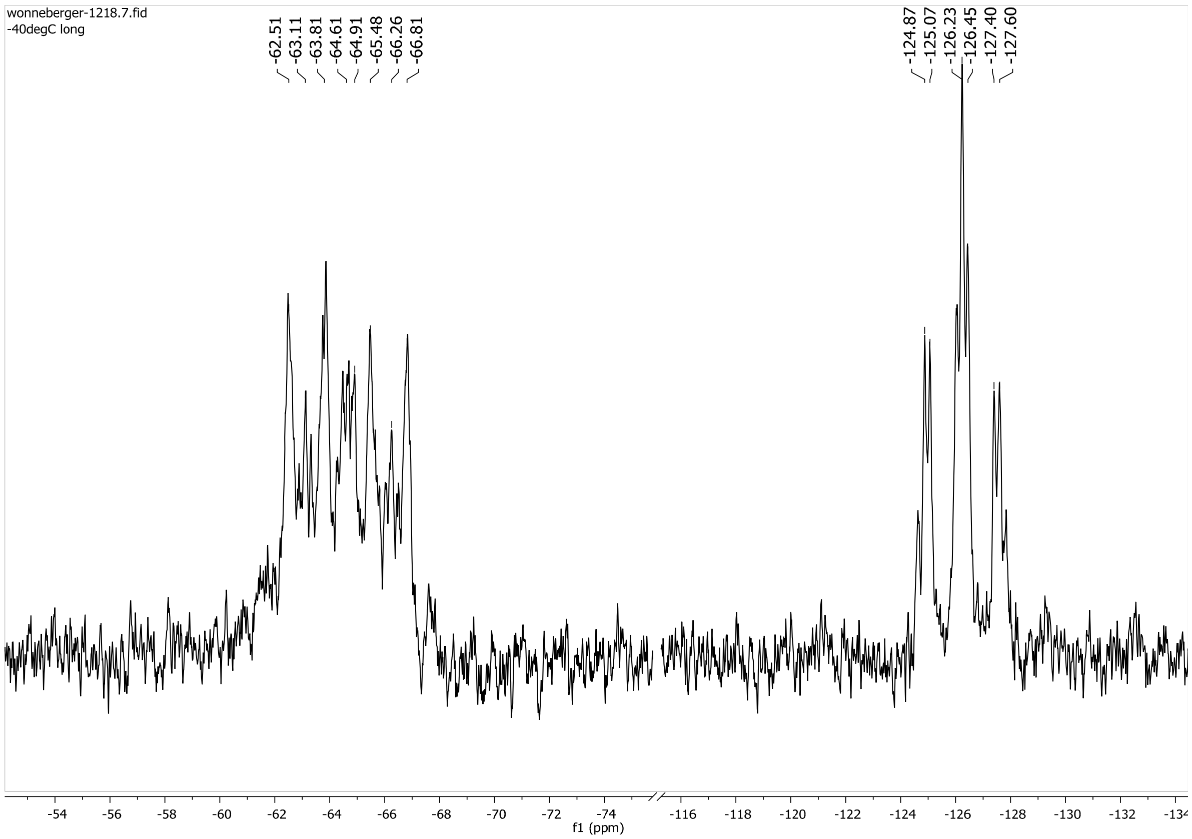

Figure S27. $^{31}P\{^{1}H\}$ NMR spectrum of complex **4** at −60 °C.

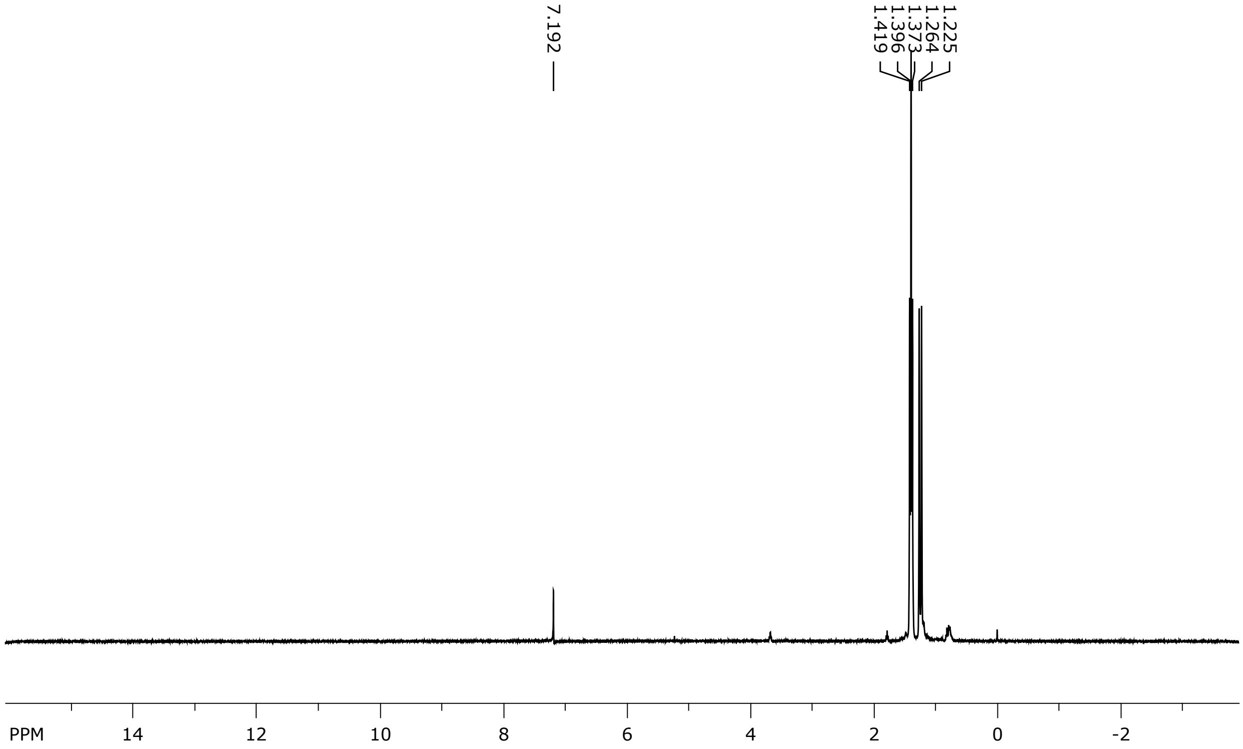

Figure S28. ^{1}H NMR spectrum of complex **5** at *room temperature*.

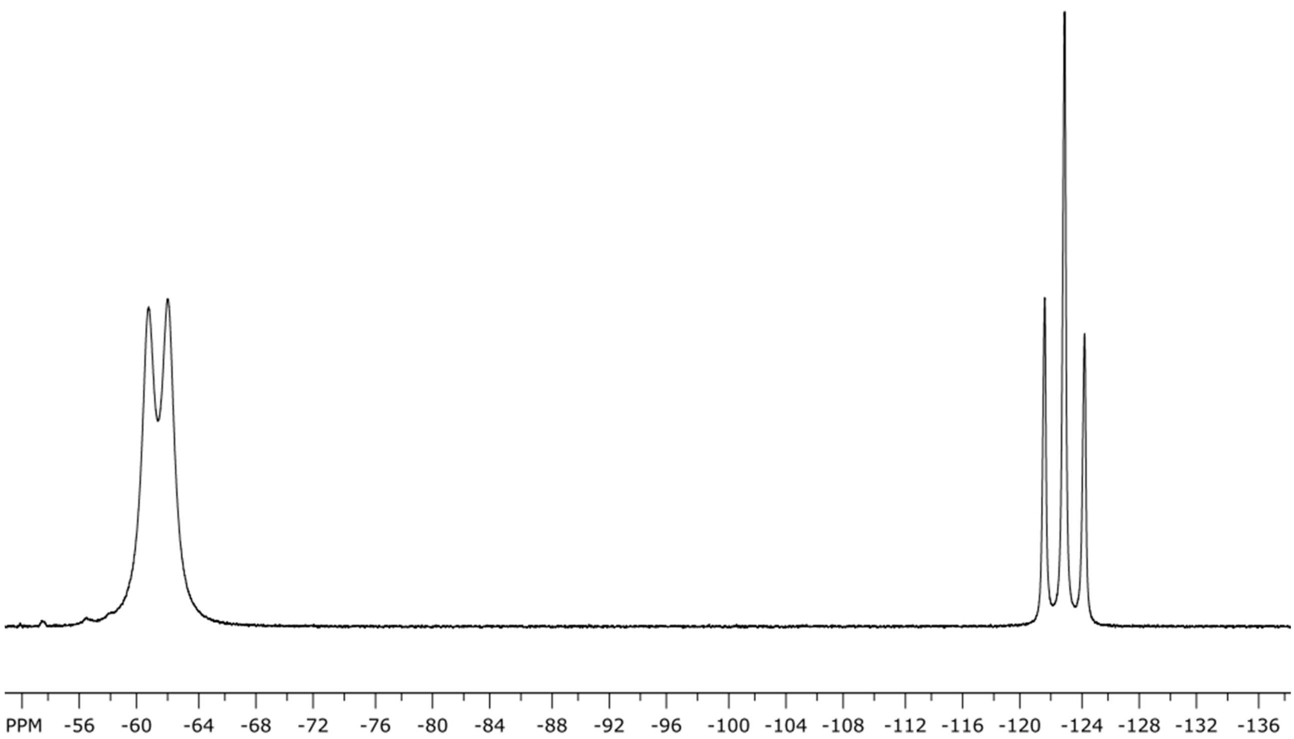

Figure S29. $^{31}P\{^1H\}$ NMR spectrum of complex **5** at *room temperature*.

6. Thermal Analysis

In order to test if the complexes containing *cyclo*-(P_3*t*Bu_3) are suitable candidates for a conversion to phosphorus-rich metal phosphides, the thermolysis of complex **2** was investigated by thermogravimetry (TG) in combination with differential thermal analysis (DTA) as well as mass spectrometry (MS) in the temperature range 30–900 °C under an argon atmosphere. The residue was investigated with PXRD. The thermolysis of [Cu_4(μ-Br)$_4${*cyclo*-(P_3*t*Bu_3)}$_2$] (**2**) occurred in four steps (Figure S30). The first and major step at about 260 °C comprised a mass loss of 54%. Coupled mass spectrometry revealed the release of *t*Bu groups in the form of *iso*-butene, as is typical for *t*Bu-substituted oligophosphorus complexes[61-63] as well as Br atoms in the form of HBr, discernable by its isotopic pattern (^{79}Br with 51% and ^{81}Br with 49% abundance)[61]. The mass loss corresponds to four *t*Bu groups and three Br atoms. The onset temperature for the release of the *t*Bu groups is observed in the same range for similar complexes.[61-63] The following step at about 420 °C comprises the loss of two *t*Bu groups. Until about 750 °C, a rather continuous mass loss is observed during which also white phosphorus (P_4) is detected by coupled mass spectrometry. The final product was analyzed by powder X-ray diffraction and could be identified as a mixture of Cu_3P and elemental Cu (Figure S31). Thus, no phosphorus-rich metal phosphide was obtained from complex **2**. Future investigations on the other complexes described here might lead to different results.

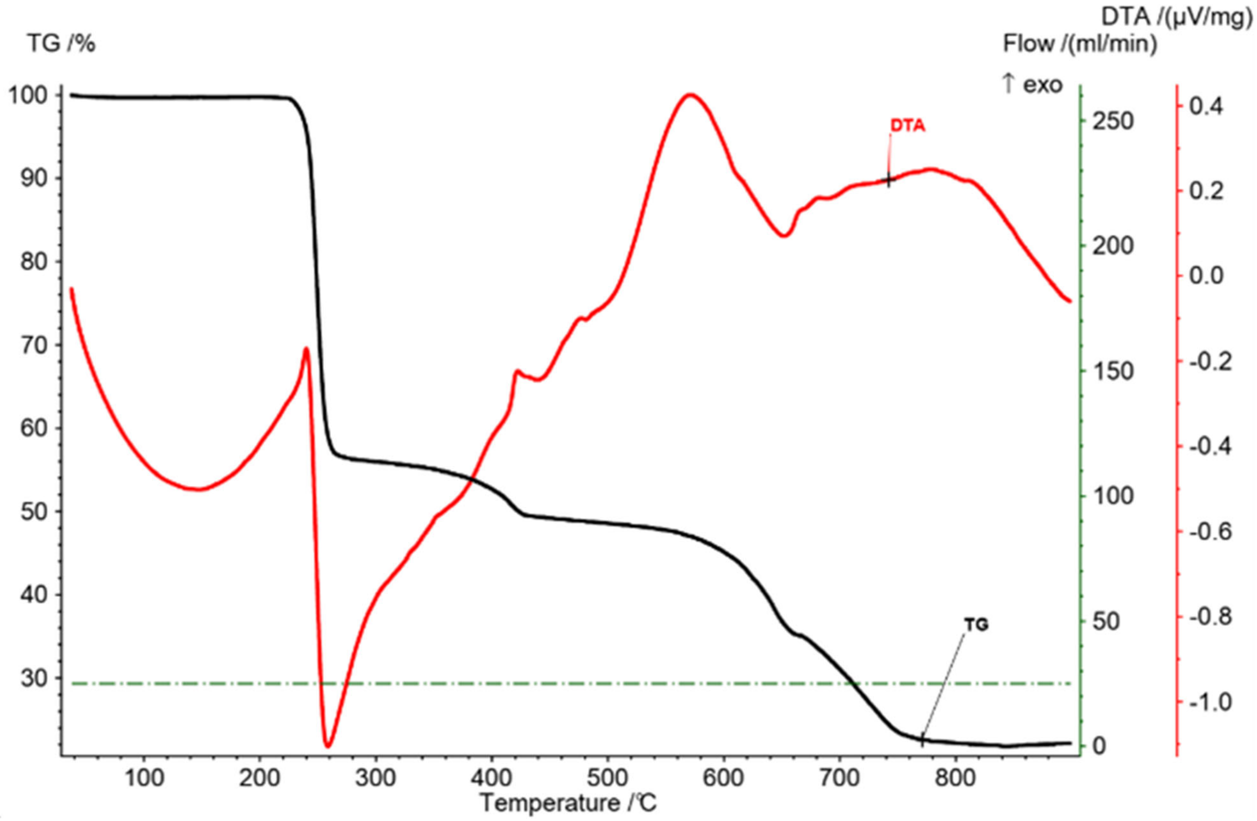

Figure S30. TG/DTA curve of complex **2**.

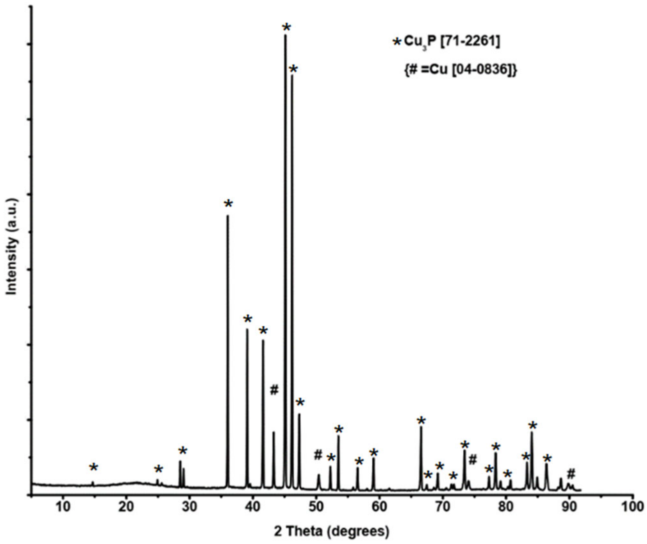

Figure S31. Powder X-ray diffractogram of the substance obtained after thermolysis of complex **2**. The two phases Cu_3P and Cu could be identified as crystalline components.

7. References

[45] Agilent (**2014**). *CrysAlis PRO*. Agilent Technologies Ltd, Yarnton, Oxfordshire, England.

[46] G. M. Sheldrick, *Acta Crystallogr., Sect. A: Found. Adv.*, **2015**, *71*, 3.

[47] G. M. Sheldrick, *Acta Crystallogr., Sect. C: Struct. Chem.*, **2015**, *71*, 3.
[48] L. J. Farrugia, *J. Appl. Crystallogr.*, **2012**, *45*, 849.
[49] H. Putz, K.Brandenburg, DIAMOND, Crystal Impact GbR, Bonn, Germany, **1999**.
[50] STOE Cie GmbH. *WinXPow*; **2014**.
[51] Topas, Bruker AXS GmbH, Karlsruhe, **2014**.
[52] C. F. Macrae, I. J. Bruno, J. A. Chisholm, P. R. Edgington, P. McCabe, E. Pidcock, L. Rodriguez-Monge, R. Taylor, J. van de Streek, P. A. Wood, *J. Appl. Crystallogr.*, **2008**, *41*, 466.
[53] M. Baudler, J. Hahn, H. Dietsch, G. Fürstenberg, *Z. Naturforsch. B,* **1976**, *37B*, 1305.
[54] F. Neese, *Wiley Interdiscip. Rev.: Comput. Mol. Sci.*, **2018**, 8.
[55] E. Caldeweyher, C. Bannwarth and S. Grimme, J. Chem. Phys., **2017**, *147*, 34112.
[56] E. Caldeweyher, S. Ehlert, A. Hansen, H. Neugebauer, S. Spicher, C. Bannwarth and S. Grimme, J. Chem. Phys., **2019**, *150*, 154122.
[57] Harris, R. K.; Becker, E. D.; Cabral De Menezes, S. M.; Goodfellow, R. J.; Granger, P. Concepts Magn. Reson. **2002**, *14*, 326.
[58] in DAISY, part of TopSpin 4.1.1, Bruker BioSpin GmbH, Rheinstetten, **2020**.
[59] J. P. Albrand, A. Cogne, J. B. Robert, J. Am. Chem. Soc. **1978**, *100*, 2600.
[60] N. Karschin, S. Krenek, D. Heyer, C. Griesinger, *Magn. Reson. Chem.*, **2022**, *60*, 203.
[61] T. Grell, E. Hey-Hawkins, *Inorg. Chem.* **2020**, *59*, 7487.
[62] A. Kircali, R. Frank, S. Gómez-Ruiz, B. Kirchner, E. Hey-Hawkins, *ChemPlusChem* **2012**, *77*, 341.
[63] A. Kircali, P. Lönnecke, E. Hey-Hawkins, *Z. Anorg. Allg. Chem.* **2014**, *640*, 271.
